# Healing Knowledge: Conservation Challenges for Medicinal Plants in Paraguay

**DOI:** 10.3390/plants15203223

**Published:** 2026-10-20

**Authors:** María I. Vera-Jiménez, Luis Delgado-Sánchez, Luz María Muñoz-Centeno

**Affiliations:** 1 Laboratorio de Recursos Vegetales, Departamento de Biología, Facultad de Ciencias Exactas y Naturales, Universidad Nacional de Asunción, San Lorenzo 111421, Paraguay; 2 Department of Botany and Plant Physiology, University of Salamanca, 37008 Salamanca, Spain; ldelsan@usal.es (L.D.-S.); luzma@usal.es (L.M.M.-C.)

**Keywords:** Paraguay, medicinal plants, traditional medicine, uses, protected species, conservation

## Abstract

Medicinal plants in Paraguay, as part of traditional medicine, are used to maintain health and/or treat specific ailments. These are utilised in many ways within both allopathic and traditional medical systems worldwide. The present study sought to analyse the species of medicinal plants utilised by the Paraguayan population. This analysis was based on publications from 1972 to 2024 and ethnobotanical interviews. The number of mentions over time, the parts of the body targeted by each species, and the plant parts used for medicinal purposes were considered. The publications were compiled from international databases, alongside data derived from ethnobotanical fieldwork. Specifically, 95 semi-structured interviews were conducted with 50 sellers (*yuyeros/as*) and users of medicinal plants in municipal markets across various cities in the country. A total of 297 species were recorded in the study, of which 140 were identified during the interviews. Then, 33 of these medicinal species are classified as Endangered (EN) or threatened with extinction (AE) by the Ministry of Sustainable Development of Paraguay (2025). This classification is indicative of the impact of population decline or restricted distribution within the country. Only 15 of these plants are on the IUCN Red List. Notable examples include *Balfourodendron riedelianum* (Engl.) Engl. and *Plectrocarpa sarmientoi* (Lorentz ex Griseb.) Christenh. & Byng, categorised as Endangered (EN); *Libidibia paraguariensis* (D. Parodi) G.P. Lewis, classified as Vulnerable (VU); and *Araucaria angustifolia* (Bertol.) Steud. is listed as Critically Endangered (CR). Appropriate conservation efforts could play a key role in preventing further deterioration.

## 1. Introduction

Traditional medicine (TM) encompasses the knowledge, skills and practices based on the theories, beliefs and experiences indigenous to different cultures, whether explicable or not, that people use to maintain health and to prevent, diagnose, improve or treat physical and mental illnesses [1].

Within TM, medicinal plants are used to maintain health and/or treat specific ailments, and practitioners employ them in many ways within allopathic and traditional medicine systems worldwide [2,3].

Paraguay has approximately 6 million inhabitants [4], divided into two natural regions, eastern and western, with most of the population concentrated in the former. Paraguayans use and consume fresh and/or dried medicinal plants, directly or in infusions, decoctions and/or macerations, as part of a strong cultural tradition.

This practice derives from the blend of indigenous Guaraní culture with that of the Spanish [5,6]. People use medicinal plant species both preventively and curatively, and many chronic conditions receive treatment with these plants. The population frequently turns to medicinal plants to treat conditions such as diabetes and hypertension, and even to regulate fertility [7,8]. In addition to the methods mentioned, people consume medicinal plants daily in large quantities in *mate* or *tereré* [7].

Recent publications have documented the country’s extensive knowledge of using plants to maintain health, including among Paraguayan immigrants in the Argentinian province of Misiones [6,7,9,10]. According to Pin et al. [7], the conservation of native medicinal plants is threatened by several factors, including excessive and unsustainable harvesting (due to poor resource management practices), lack of cultivation, destruction of natural habitats, the effects of deforestation, loss of biological and cultural diversity, environmental pollution and an unchecked increase in demand from laboratories and exports, without oversight by law enforcement authorities.

Paraguay has signed and ratified the CBD (Convention on Biological Diversity), committing to the effective and efficient implementation of programmes related to conservation, ecological restoration and the sustainable use of biodiversity. The commitment also includes institutional strengthening and national and international legal frameworks, with the principles of the CBD and the rights of indigenous peoples with regard to traditional knowledge given prominence, in an effort to prevent biopiracy. However, it has not yet signed the Nagoya Protocol [11].

At the national level, in 2025, the Ministry of Sustainable Development (MADES) published Resolution 512/25 [12], which updates the list of protected species of native wild flora in Paraguay. It has been determined that species categorised by the IUCN as “endangered, critically endangered and data deficient” correspond to the MADES category “in danger of extinction”, and those categorised as “vulnerable” correspond to the MADES category “threatened with extinction”.

In addition, despite the extensive and specific nature of traditional knowledge, there are still species used in traditional medicine that present problems of taxonomic identity. This is probably due to the fact that the same common name is used to designate different species that are used for the same purpose.

Based on the background information provided, the aim of this study was to analyse the literature on medicinal plants in Paraguay and, through an ethnobotanical survey of key markets across the country, to identify the plants on sale, their traditional uses, and the conservation status of the species identified. The overall aim was to contribute to the understanding, appreciation, and biocultural conservation of these natural resources.

## 2. Results and Discussion

### 2.1. Diversity of Medicinal Plants in the Paraguayan Population: Medicinal Uses Prospection (Bibliographic Review)

In the review process, 297 species were compiled, of which 251 have three or more mentions in the various bibliographic references consulted (not including duplicate information). In addition, 46 species with one or two mentions are considered nationally threatened (Table 1).

The 12 most frequently mentioned native species were: *Solanum sisymbriifolium* Lam. (ñuati pytá) (13), *Dysphania ambrosioides* (L.) Mosyakin & Clemants (ka’a re) (12), *Sida cordifolia* L. (malva blanca) (12), *Piper regnellii* (Miq.) DC. (jaguarundí) (12), *Acrocomia aculeata* (Jacq.) Lodd. ex Mart. (mbocajá) (11), *Acanthospermum hispidum* D.C. (tororatí) (11), *Baccharis trimera* (Less.) DC. (jaguareté ka’a) (11), *Xanthium spinosum* L. (cepacaballo) (11), *Begonia cucullata* Willd. (agrial) (11), *Maytenus ilicifolia* Mart. ex Reissek Biral (kangorosa) (11), *Dorstenia brasiliensis* Lam. (taropé) (11), and *Cecropia pachystachya* Trécul (amba’y) (11).

In addition, a total of 89 botanical families were recorded, and the 22 with more than four species were Asteraceae (42), Fabaceae (25), Lamiaceae (11), Poaceae (10), Amaranthaceae (9), Malvaceae (9), Rutaceae (9), Verbenaceae (9), Myrtaceae (8), Bignoniaceae (7), Cucurbitaceae (7), Euphorbiaceae (6), Solanaceae (6), Anacardiaceae (5), Apiaceae (5), Moraceae (5), Urticaceae (5), Apocynaceae (4), Lauraceae (4), Lythraceae (4), Pteridaceae (4) and Santalaceae (4), which together account for 67% of the species. The remaining 67 families have 1–3 species and account for 33%.

Among the botanical families that contribute most to traditional medicine, Asteraceae (42) and Lamiaceae (11) almost always stand out among the top families, which is consistent with Tello-Cerón [13], Martínez Moreno [14], Angulo [15], De la Torre [16], and De la Cruz [17] for different countries in the Americas. This prominence can be attributed to the abundance of species in these two families that contain active compounds (secondary metabolites–bioactive substances) that have been utilised by humans [18].

### 2.2. Ethnobotanical Survey

**Figure 1:**
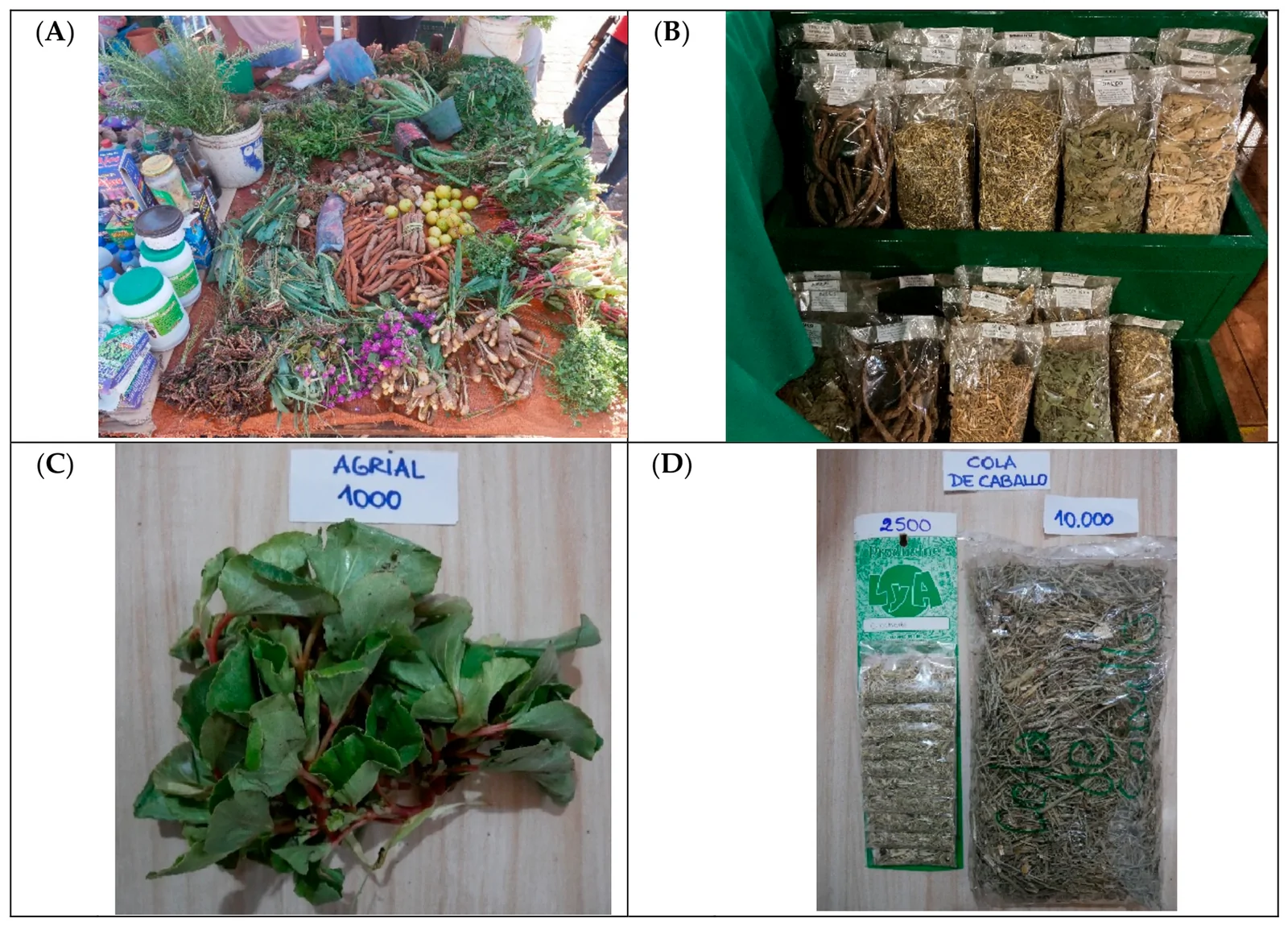

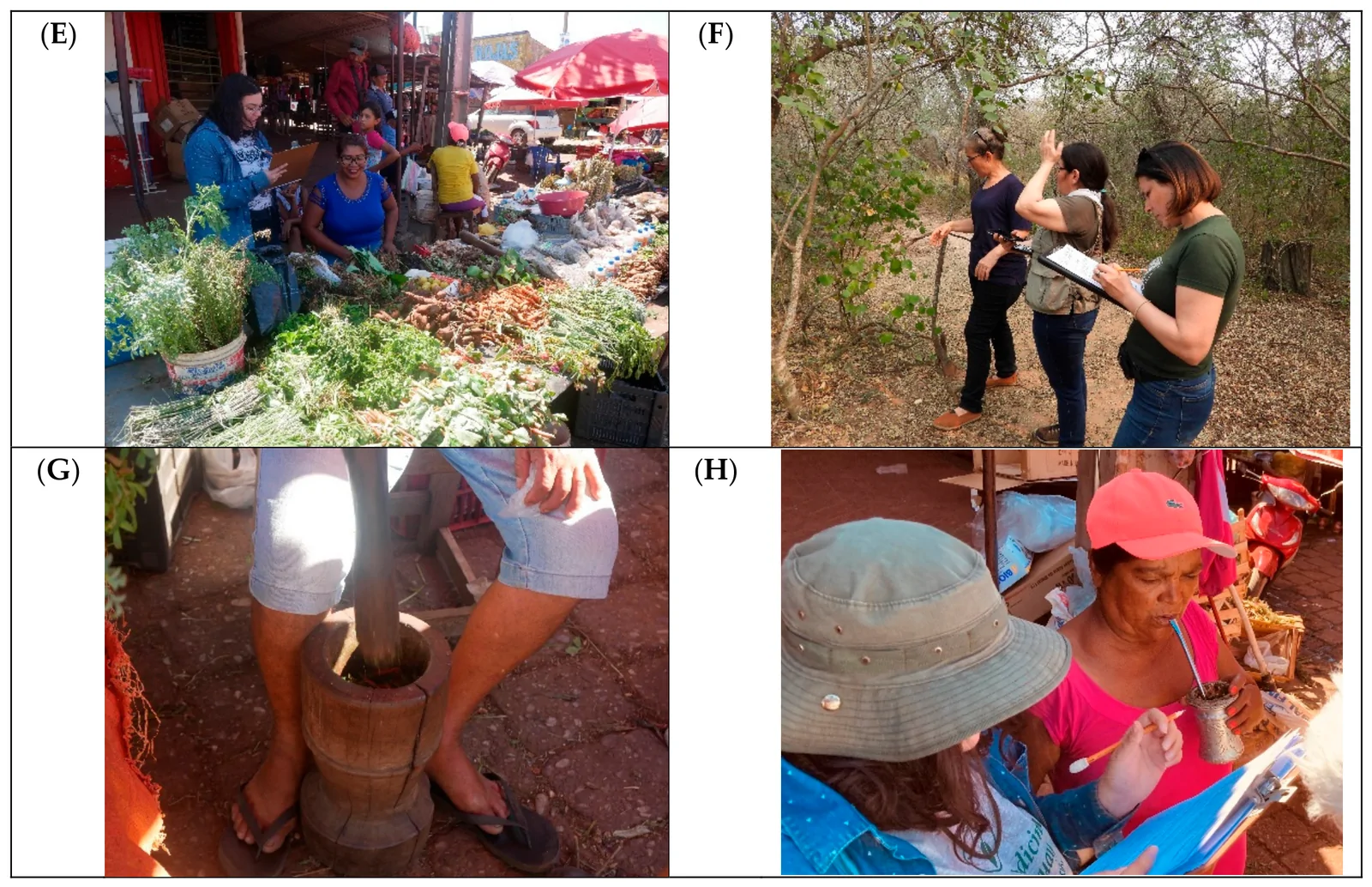
(**A**). Fresh medicinal species. (**B**). Dried medicinal plants packaged for sale. (**C**). Agrial (*Begonia cucullata*) sold fresh with their prices in local currency (guaraníes). (**D**). Cola de caballo (*Equisetum giganteum*) sold dried with their prices in local currency (guaraníes). (**E**). Interview at a market stall in the Eastern Region (Concepción). (**F**). Interview at a municipal reserve in the Western Region (Neuland). (**G**). Crushing fresh species for tereré water. (**H**). Drinking tereré.

A total of 140 species were recorded during the course of the interviews (Figure 1E,F), 139 of which correspond to those mentioned in the bibliographic references. Among the species under consideration, *hercampure* (*Gentianella alborosea* (Gilg.) Fabris) is worthy of particular note; it is an imported medicinal species that has been used to treat disorders of the digestive, circulatory, and endocrine–metabolic systems. However, it is not mentioned in the cited references.

In Paraguay, the utilisation of traditional botanical knowledge for therapeutic purposes is deeply entrenched in daily practices. This is exemplified by the consumption of *mate*, a traditional beverage consumed early in the morning when temperatures are low, and *tereré*, a beverage consumed during the day when temperatures rise.

The frequency and diversity of species utilised can be attributed to the practice of incorporating herbs and medicinal plants into cold (*tereré*) or hot (*mate*) water preparations. The resulting mixture or solution is then measured and poured into portions of 20 to 40 mL, which are subsequently consumed in the traditional *mate*/*matero* and/or *guampa* (Figure 1G).

The freshly prepared brew [19] is consumed using a *bombilla* (metal straw) [20] until the desired temperature of the brew is reached, which can range from 1 to 2 litres (Figure 1H).

The plants added to the water can have different origins; of the 297 species reviewed, 64 are introduced, naturalised or cultivated. Examples of cultivated species include *Borago officinalis* (*borraja*), *Salvia rosmarinus* (*romero*), *Sambucus australis* (*sauco*) or *Laurus nobilis* (*laurel de España*), native to Europe; or *Nicotiana tabacum* (*tabaco*), *Cucurbita máxima* (*zapallo*), *Carica papaya* (*papaya*) or *Aloe vera* (*aloe*), native to North and Central America.

Species such as *ruda* (*Ruta graveolens*), *borraja* (*Borago officinalis*), and *agosto poty* (*Senecio grisebachii*) contain active compounds that may be toxic [7].

It is noteworthy that 15 species used medicinally in Paraguay are not listed in the Medicinal Plant Names Services (MPNS) database [21]. These species are: *Annona cacans, Cohniella jonesiana*, *Hemionitis rufa*, *Citharexylum myrianthum*, *Couepia grandiflora*, *Lithachne pauciflora*, *Ocimum ovatum*, *Peperomia circinnata*, *Pereskia nemorosa*, *Rhynchosia edulis*, *Scleria distans*, *Senecio grisebachii*, *Sidastrum paniculatum*, *Trimezia spathata* and *Viguiera linearifolia*.

**Table 1:** Medicinal plants used by the Paraguayan population ([5,6,7,8,9,10,22,23,24,25,26,27,28,29,30,31,32,33,34,35,36,37,38]). References: Body system: SC: circulatory system, SD: digestive system, SG-U: genitourinary system, SR: respiratory system, SE-M: endocrine–metabolic system, ST: integumentary system, SN: nervous system, SM-E: musculoskeletal system. Origin: N: native, I: introduced, Nat: naturalised. * Medicinal species included in a category of MADES protection [12,39]. Grey shading: medicinal species recorded in the interviews for this study. Bold: new record.

Family	Scientific Name	Vernacular Name	Habit	Part Used	Body System	Origin	Citations
Amaranthaceae	*Alternanthera brasiliana* (L.) Kuntze	penicilina	subshrub	fresh or dried aerial part	ST	N	3
Amaranthaceae	*Alternanthera ficoidea* (L.) P. Beauv.	perdudilla negra	perennial herb	leaves	SG-U, SN	N	3
Amaranthaceae	*Alternanthera pungens* Kunth	ñuati pe, hierba de pollo	herb	whole plant	SD, SC, SG-U, SM-E, ST	N	7
Amaranthaceae	*Amaranthus muricatus* (Moq.) Hieron.	hierba meona	herb	aerial part	SC, SG-U, ST	N	2
Amaranthaceae	*Dysphania ambrosioides* (L.) Mosyakin & Clemants	ka’a rë, paico, wayketom, wayke aktom	suffrutescent herb	aerial part, seeds	SD, SG-U, SR, SC, ST	Nat	12
Amaranthaceae	*Gomphrena celosioides* Mart.	perdudilla blanca, paludilla	perennial herb	whole plant	SR, SD, SC, SG-U, SE-M	N	8
Amaranthaceae	*Gomphrena perennis* L.	siempre viva	perennial herb	flowers and aerial parts	SC, SN, SE-M, SG-U	N	3
Amaranthaceae	*Iresine diffusa* Humb. & Bonpl. ex Willd.	mbói ka’a	herb	flowering plant	SE-M, SD, SG-U	N	3
Amaranthaceae	*Pfaffia glomerata* (Spreng.) Pedersen*	batatilla	herb	fresh tubers	SD, SC, ST	N	4
Anacardiaceae	*Mangifera indica* L.	mango	tree	leaves, fruit	SD, SE-M	I	5
Anacardiaceae	*Myracrodruon urundeuva* Allemão	urunde’y mi	tree	bark, dried leaves	SC, SD	N	3
Anacardiaceae	*Schinopsis lorentzii* (Griseb.) Engl.	coronillo, quebracho colorado, tujni	tree	wood	SD, ST, SG-U, SN	N	3
Anacardiaceae	*Schinus weinmannifolia* Engl.	molle’i	tree/shrub	aerial part	SE-M, SR, SG-U	N	8
Anacardiaceae	*Schinus terebinthifolia* Raddi	aguaray o molle-mi	tree	resin, leaves, branches, fruits	ST, SM-E	N	4
Anemiaceae	*Anemia tomentosa* (Savigny) Sw. var. *tomentosa*	amambai-akuä, doradilla aromática	fern	aerial part	ST, SG-U, SR, SD	N	4
Annonaceae	*Annona emarginata* (Schltdl.) H.Rainer	aratikú’i	tree	leaves	SR, SE-M	N	5
Annonaceae	*Annona muricata* L.	corazón de india	tree	leaf	SE-M, SD, SN	I	3
Annonaceae	*Annona cacans* Warm. *	aratikú guasú	tree	seeds, leaves	ST, SR	N	1
Apiaceae	*Anethum graveolens* L.	eneldo	herb	fruit, seed, bark and root	SD, SN	I	5
Apiaceae	*Coriandrum sativum* L.	coriandro, kuratú	subshrub	whole plant, seeds	SD, SN	I	3
Apiaceae	*Foeniculum vulgare* Mill.	hinojo	herb	fruits	SD, SC	I	6
Apiaceae	*Petroselinum crispum* (Mill.) Fuss	perejil	herb	root	SG-U, SD, SR	I	3
Apiaceae	*Pimpinella anisum* L.	anis	herb	fruit, seed	SD, SN, SR	I	4
Apocynaceae	*Aspidosperma quebracho-blanco* Schltdl.	quebracho blanco, ebedu	tree	bark	SD, ST, SC, SR, SN	N	9
Apocynaceae	*Mandevilla angustifolia* (Malme) Woodson		vine	tuberous root	SG-U	N	1
Apocynaceae	*Rauvolfia sellowii* Müll. Arg. *	quina, pseudoquina, palo amargo, kino	tree	bark	SE-M, SD	N	1
Apocynaceae	*Tabernaemontana catharinensis* A. DC.	sapirangy	shrub/tree	dried leaves, latex	SD, ST	N	3
Aquifoliaceae	*Ilex paraguariensis* A. St.-Hil.	ka’a, yerba mate	tree	leaves, branches	SD, SC	N	5
Araliaceae	*Hydrocotyle bonariensis* Lam.	akäryso	herb	whole plant, leaves	SD, ST	N	3
Araliaceae	*Hydrocotyle leucocephala* Cham. & Schltdl.	san antorio sombrero, akaryso	herb	whole plant	ST, SD, SG-U	N	4
Araucariaceae	*Araucaria angustifolia* (Bertol.) Steud. *	kuri’y	tree	resin (balsam)	ST, SD	N	1
Arecaceae	*Acrocomia aculeata* (Jacq.) Lodd. Ex Mart.	mbokajá, cocotero, coco’i	palm	seedling, root, leaves, fruit, seed	SC, SD, SG-U, SM-E, SN	N	11
Arecaceae	*Syagrus romanzoffiana* (Cham.) Glassman	pindó	palm	dried root of young plant	SC, SG-U, SD, SE-M, SM-E	N	8
Aristolochiaceae	*Aristolochia triangularis* Cham.	ysypo mil hombre	vine	stems, roots	SG-U, SD, SM-E, SN, ST	N	9
Asparagaceae	*Herreria montevidensis* Klotzsch ex Griseb. *	zarzaparrilla, ysypó mandi’o, pongwanay, jotekatade	vine	root	SD, SG-U, SC, SM-E	N	9
Asphodelaceae	*Aloe vera* (L.) Burm. f.	aloe	herb	leaf	SD, SG-U	I	5
Asteraceae	*Acanthospermum australe* (Loef.) Kuntze	tapekue	herb	whole plant	ST, SD, SM-E, SG-U	N	9
Asteraceae	*Acanthospermum hispidum* DC.	tororatí, kapiatï	annual herb	fresh leaves	SR, SC, SD, SG-U	N	11
Asteraceae	*Achillea millefolium* L.	mitakuña rague, mil flores, mil hojas	suffrutescent herb	flowers, leaves	SC, SD, SG-U, SN	I	5
Asteraceae	*Achyrocline alata* (Kunth) DC. *	jate’i ka’a	suffrutescent herb	dried plant, leafy-flowering stems	SD, SG-U, SE-M, SM-E, SR	N	9
Asteraceae	*Achyrocline satureioides* (Lam.) DC./*Achyrocline saturejoides* (Lam.) DC.	marcela, marcelita	herb	flowering top	SM-E, SD, ST	N	8
Asteraceae	*Ambrosia artemisiifolia* L.	altamisa, hierba Santa María	herb	young leaves and stems	SG-U, ST, SD	N	6
Asteraceae	*Ambrosia tenuifolia* Spreng.	altamisarä	herb	aerial part	SG-U, SD, SN	N	5
Asteraceae	*Artemisia absinthium* L.	ajenjo	subshrub	aerial part	SD, SG-U	Nat	6
Asteraceae	*Austroeupatorium inulifolium* (Kunth) R.M. King & H. Rob.	doctorcito, sanalotodo	herb	aerial part	SD, SC, ST, SG-U	N	9
Asteraceae	*Baccharis articulata* Pers.	chirca melosa, carqueja dulce	subshrub	whole plant	SD, SR, SC, ST, SE-M,	N	7
Asteraceae	*Baccharis crispa* Spreng. *	jaguareté ka’a	suffrutescent herb	aerial part	SG-U, SD, SE-M	N	3
Asteraceae	*Baccharis microcephala* (Less.) DC. *	jaguareté ka’a, carqueja	subshrub	flowering tops	SG-U, SD	N	2
Asteraceae	*Baccharis trimera* (Less.) DC. *	jaguarete ka’a, carqueja	subshrub	aerial part, stems, leaves	SD, SG-U, SM-E, ST	N	11
Asteraceae	*Bidens pilosa* L.	kapi’una	annual herb	aerial part	SG-U, SC, SD	N	9
Asteraceae	*Carthamus tinctorius* L.	azafrán, arazó, azafrán paraguayo	herb	stigmas	SD, SN, SG-U	I	7
Asteraceae	*Chaptalia nutans* (L.) Pol	lengua de buey, cerraja	perennial herb	leaves, roots	SM-E, ST, SG-U, SC	N	5
Asteraceae	*Chrysolaena cognata* (Less.) Dematteis	jagua ra’yi	herb	whole plant, rhizome	SE-M, SC	N	4
Asteraceae	*Cyclolepis genistoides* D.Don *	palo azul	shrub	aerial part	SD, SM-E, SC, SE-M	N	3
Asteraceae	*Elephantopus mollis* Kunth	azuá-azuá, chicoria, fumo bravo, llantén hú	herb	leaves, aerial part	SR, SE-M	N	3
Asteraceae	*Gamochaeta americana* (Mill.) Wedd.	vira vira	annual herb	whole plant	SD, SG-U, SE-M	N	3
Asteraceae	*Moquiniastrum polymorphum* (Less.) G.Sancho	tataré moroti, kambará	tree	fresh leaves	SR	N	9
Asteraceae	*Hypochaeris sessiliflora* Kunth	ka’apé, achicoria del campo	herb	leaves, whole plant (without flowers)	SD, SC	N	6
Asteraceae	*Hypochaeris chillensis* (Kunth) Britton	achí, achicoria, chicoria	herb	young leaves and root	SD, SR, SG-U	N	3
Asteraceae	*Jungia floribunda* Less.	jaguarete po	herb	root, leaves	SE-M, SC, SR, ST	N	3
Asteraceae	*Matricaria chamomilla* L.	manzanilla	herb	inflorescences	SD, SM-E, ST	I	4
Asteraceae	*Pectis odorata* Griseb.	ciervo ka’a, guasú ka’a, manzanilla del campo	annual herb	whole plant	SD, SR	N	4
Asteraceae	*Pluchea sagittalis* (Lam.) Cabrera	yerba de lucero, kito	perennial herb	fresh or dried aerial part	SD, SR, ST	N	10
Asteraceae	*Porophyllum lanceolatum* DC.	kurupa’y mi	annual herb	dried plant	SR, SC, SE-M, ST	N	5
Asteraceae	*Porophyllum ruderale* (Jacq.) Cass.	yryvu retyma	annual or perennial herb	fresh or dried aerial part	SM-E, SD, ST, SN	N	6
Asteraceae	*Pterocaulon polystachyum* DC.	toro ka’a	perennial herb	dried plant with root	SC, SD, SG-U	N	3
Asteraceae	*Pterocaulon virgatum* (L.) DC.	toro ka’a moroti, tujá kasó, kelkaalik yaamit	perennial herb	aerial part, leaves, fresh plant	SR, SD, ST	N	5
Asteraceae	*Schkuhria pinnata* (Lam.) Kuntze ex Thell.	canchalagua’i	herb	aerial parts	SG-U, SD, SC, SE-M, SN	N	4
Asteraceae	*Senecio grisebachii* Baker	agosto poty, flor de agosto	herb	aerial part	SC	N	5
Asteraceae	*Solidago chilensis* Meyen	eupatorio, mbuy guasú, mbuy say ju, typychá moroti, cohete ruguay	suffrutescent herb	aerial part, flowers, seeds	SC, SD, SG-U, SM-E, SE-M	N	5
Asteraceae	*Sonchus oleraceus* L.	Cerraja	annual herb	aerial part	SD, SG-U, SC, SE-M	N	4
Asteraceae	*Stevia entreriensis* Hieron. ex Arechav.	charrúa ka’a	annual herb	leaves	SD	N	6
Asteraceae	*Stevia rebaudiana* (Bertoni) Bertoni *	ka’a he’é	herb	leaves and dried flowering stems	SE-M, SD, SC	N	9
Asteraceae	*Tagetes minuta* L.	Suico	annual herb	fresh plant without flowers, aerial part	SD, SG-U, SN	N	6
Asteraceae	*Trixis pallida* Less.	urusu katí	herb	root	SD, SN	N	4
Asteraceae	*Vernonanthura tweedieana* (Baker) H. Rob.	jagua pety	shrub	dried rhizome	SC, ST	N	3
Asteraceae	*Viguiera linearifolia* Chodat	arnica del campo	herb	root	SC, SD	N	2
Asteraceae	*Xanthium spinosum* L.	cepacaballo, abrojito, nooling aptom	annual herb	aerial part, root	SC, SD, SG-U, SR	N	11
Begoniaceae	*Begonia cucullata* Willd.	agrial, agrial pytä	herb	fresh aerial parts, root	SR, SD, ST, SE-M	N	11
Bignoniaceae	*Adenocalymma marginatum* (Cham.) DC.	ysypo hu	vine	root	SE-M, SG-U, SD	N	3
Bignoniaceae	*Dolichandra unguis-cati* (L.) L.G. Lohmann	mbarakajá pyapé	liana	fresh or dried root	SG-U, SM-E, SE-M	N	8
Bignoniaceae	*Handroanthus heptaphyllus* (Vell.) Mattos *	tajy hú	tree	bark, flowers	ST, SR	N	2
Bignoniaceae	*Handroanthus impetiginosus* (Mart. ex DC.) Mattos	tajy pytä	tree	bark, bark powder	SD, SE-M, SC	N	4
Bignoniaceae	*Jacaranda micrantha* Cham.	Karoba	tree	bark, stem	SG-U, SD, SC, ST	N	1
Bignoniaceae	*Jacaranda mimosifolia* D. Don	Jacaranda	tree	leaves, bark	SG-U, ST, SE-M	N	5
Bignoniaceae	*Tabebuia aurea* (Silva Manso) Benth. & Hook. f. ex S. Moore	Paratodo	tree	dried bark	SM-E, SR, SD, SE-M, ST	N	8
Bixaceae	*Bixa orellana* L.	urukú	tree	seeds	ST	N	5
Bixaceae	*Cochlospermum regium* (Schrank.) Pilg.	mandyju’i	shrub	root	SR, SM-E, SD, SG-U	N	1
Boraginaceae	*Borago officinalis* L.	borraja	herb	flowers	SR, ST, SE-M	I	7
Bromeliaceae	*Bromelia balansae* Mez	karaguatá	herb	fruits, root	SD, SC, SG-U, SE-M	N	5
Bromeliaceae	*Tillandsia recurvata* (L.) L.	flor del aire	epiphytic herb	flowers	SN, SD, SE-M	N	1
Bromeliaceae	*Tillandsia usneoides* (L.) L.	clavel del aire, barba de viejo	epiphytic herb	leaves, stems	SD, SE-M, ST, SC, SM-E	N	1
Burseraceae	*Protium heptaphyllum* (Aublet) Marchand	ysy	tree	resin (balsam), bark, leaves	ST, SN, SD, SR, SM-E, SE-M	N	6
Cactaceae	*Harrisia bonplandii* (Parm. ex Pfeiff.) Britton & Rose	reina de noche, pasacana	shrub	stems, flowers	SN, SC, SM-E	N	1
Cactaceae	*Pereskia nemorosa* Rojas Acosta	amapola	small tree	seeds and flowering tops	SE-M, SM-E, SD, SN, SR	N	3
Cactaceae	*Pereskia sacharosa* Griseb.	amapola	small tree	seeds and flowering tops	SM-E, SD, SR, SN	N	1
Calyceraceae	*Acicarpha tribuloides* A. L. Juss.	manzanilla pochy, toro rati	herb	aerial part	SG-U, SR	N	4
Cannabaceae	*Celtis iguanaea* (Jacq.) Sarg.	juasy’y	tree	dried or fresh leaves	SR, SD	N	4
Capparaceae	*Morisonia speciosa* (Griseb.) Christenh. & Byng	pajaguá naranja, bola verde, najnuru	tree	leaves, bark	SC, SD, SN, SM-E, ST	N	4
Capparaceae	*Morisonia tweedieana* (Eichler) Christenh. & Byng	hoja redonda, guioatua	shrub	leaves	SD, SM-E, SG-U, ST, SR	N	2
Caprifoliaceae	*Valeriana officinalis* L.	valeriana, ka’aimbe-mi	suffrutescent herb	root, flowers	SN	I	3
Caricaceae	*Carica papaya* L.	mamón, mamonero	tree	fruit, flowers, seeds	SD, SR	Nat	8
Celastraceae	*Maytenus ilicifolia* Mart. ex Reissek *	kangorosa, sombra de toro	shrub	branches, fresh leaves, root, whole plant	ST, SD, SG-U, SC, SR	N	11
Chrysobalanaceae	*Couepia grandiflora* (Mart. & Zucc.) Benth. ex Hook. f.	surubina	tree	root	SG-U, SE-M	N	2
Clusiaceae	*Garcinia brasiliensis* Mart.	pakurí	tree	leaf	SE-M	N	3
Commelinaceae	*Commelina erecta* L.	santa Lucia hovy, hierba de santa Lucia, haape ningye	perennial herb	flowers and leaves. Whole plant	ST, SC, SG-U, SD, SE-M,	N	9
Commelinaceae	*Commelina platyphylla* Klotzsch	santa lucia moroti	annual herb	root and mucilage of floral bracts	SC, SD, ST, SG-U	N	6
Convolvulaceae	*Cuscuta* sp.	cabello de angel	parasitic herb	stems and flowers	SD, SC, SR	N	4
Convolvulaceae	*Dichondra repens* J.R. Forst. & G. Forst.	mbarakajá nambí, oreja de gato	herb	aerial part	SC, ST, SN, SR	N	6
Convolvulaceae	*Ipomoea carnea* subsp. *fistulosa* (Mart. Ex Choisy) D. F. Austin	mandyjurá	subshrub	root	SG-U, SC, SD, SE-M	N	3
Cordiaceae	*Cordia ecalyculata* Vell.	colita	tree	leaves	SC, SN	N	4
Costaceae	*Costus arabicus* L.	caña brava	aquatic-marsh herb	rhizomes, root	SC, SD, SG-U, ST, SM-E	N	8
Cucurbitaceae	*Cayaponia bonariensis* (Mill.) Mart. Crov.	tajujá moroti	creeping or climbing herb	leaves, tuber, root	SE-M, SR, SG-U, SM-E, SD	N	7
Cucurbitaceae	*Cayaponia espelina* (Silva Manso) Cogn.	aspirina del campo, tayuyá	vine	root	SD, SN, SG-U, SE-M	N	3
Cucurbitaceae	*Cucumis melo* L.	melón	vine	seeds	SD	I	3
Cucurbitaceae	*Cucurbita maxima* Duchesne	zapallo, kurapepé	vine	seeds	SD	I	4
Cucurbitaceae	*Lagenaria siceraria* (Molina) Standl.	ka’yguá, hy’a, duchubire	vine	fruits, leaves	SC, ST, SE-M	Nat	3
Cucurbitaceae	*Momordica charanthia* L.	calabacita	climbing herb	fresh aerial parts	SD, SE-M	N	6
Cucurbitaceae	*Sicana odorifera* (Vell.) Naudin	kuruguá	vine	fruit	SD	N	3
Cyperaceae	*Cyperus giganteus* Vahl	piri guasu	herb	fresh rhizome	SE-M, ST	N	1
Cyperaceae	*Cyperus sesquiflorus* (Torr.) Mattf. & Kük. subsp. *sesquiflorus*	kapi’i kati, esquenanto menor	herb	rhizome, root	SD, SG-U	N	4
Cyperaceae	*Scleria distans* Poir.	kapi’i kati, kapi’i kati estero	herb	rhizome, leaves	SD, SE-M	N	3
Dioscoreaceae	*Dioscorea campestris* Griseb.	mecho aka	vine	root	SE-M	N	3
Dioscoreaceae	*Dioscorea subhastata* Vell.	mecho aka	vine	root	SE-M	N	3
Equisetaceae	*Equisetum giganteum* L. *	cola de caballo, kavajú ruguái	herb	sterile stems	SC, SR, ST, SG-U	N	8
Euphorbiaceae	*Croton didrichsenii* G.L. Webster	bitter del campo	subshrub	root	SN, SC, SM-E, SD	N	1
Euphorbiaceae	*Croton urucurana* Baill.	sangre de drago	tree	leaves, latex, bark	SE-M, ST, SD	N	10
Euphorbiaceae	*Euphorbia lasiocarpa* Klotzsch	hierba de paloma	herb	aerial part	SC, SD, SG-U, ST, SE-M,	Nat	3
Euphorbiaceae	*Euphorbia serpens* Kunth	tupasy kamby, hierba de la golondrina, leche tresna	herb	whole plant, root	SC, SD, SG-U, ST	N	7
Euphorbiaceae	*Jatropha isabelliae* Müll. Arg.	jaguarova	herb	sterile aerial part, root	SD, SE-M, SG-U, SC	N	6
Euphorbiaceae	*Sapium haematospermum* Müll. Arg.	kurupika’y, lecherón, pega pega	tree	bark, leaves, latex	SE-M, ST, SD	N	6
Fabaceae	*Anadenanthera colubrina* (Vell.) Brenan	kurupa’y kuru	tree	bark, seeds	ST, SN	N	3
Fabaceae	*Apuleia leiocarpa* (Vogel) J.F. Macbr. *	yvyrá peré	tree	bark	SD	N	1
Fabaceae	*Bauhinia forficata* Link	pata de buey, tu’i apyngua	tree	fresh leaves	SD, SG-U, SE-M, SR, SC, ST	N	6
Fabaceae	*Cajanus cajan* (L.) Huth	kumandá yvyra’i	shrub	leaves, flowers	SR	I	7
Fabaceae	*Parkinsonia praecox* (Ruiz & Pav.) Hawkins	verde olivo, brea	shrub	exudate, tar	SR, SD	N	1
Fabaceae	*Copaifera langsdorffii* Desf.	kupa’y, copaiba	tree	oil, bark	SR, ST	N	4
Fabaceae	*Dimorphandra mollis* Benth. *	barbatimón, lorito pysa	tree	root	SD	N	1
Fabaceae	*Macropsychanthus violaceus* (Mart. ex Benth.) L.P.Queiroz & Snak	kuruguái	liana	seeds	SD, SG-U, SE-M, SM-E	N	3
Fabaceae	*Erythrina crista-galli* L.	ceibo, suinandy	tree	bark, dried leaves	SN, ST, SR	N	8
Fabaceae	*Gleditsia amorphoides* (Griseb.) Taub.	yvope	tree	bark, leaf, thorns and fruit	SN, ST, SR, SG-U	N	4
Fabaceae	*Hymenaea courbaril* L.	jata’yva	tree	bark, leaves, fruits, roots	SE-M, SD, SC	N	3
Fabaceae	*Inga affinis* DC.	inga guasu	tree	seed powder	SN	N	3
Fabaceae	*Libidibia paraguariensis* (D. Parodi) G.P. Lewis *	guayacán, carujnangue	tree	bark	SM-E, SG-U, SE-M, SD	N	4
Fabaceae	*Medicago sativa* L.	alfalfa	suffrutescent herb	aerial part	SD	I	3
Fabaceae	*Myrocarpus frondosus* Allemao *	yvyrá paje, ajuy ñandy	tree	Bark	ST, SE-M, SR, SG-U	N	3
Fabaceae	*Neltuma alba* (Griseb.) C.E. Hughes & G.P. Lewis	algarrobo blanco	tree	fruits, seeds, bark, leaves	SD, SC, SR, ST, SG-U	N	3
Fabaceae	*Neltuma kuntzei* (Harms ex Kuntze) C.E. Hughes & G.P. Lewis	palo mataco, itin, karandá, ajnutata	tree	fruits	SD, SN, SE-M	N	3
Fabaceae	*Neltuma nigra* (Griseb.) C.E. Hughes & G.P. Lewis	algarrobo negro	tree	fruits, seeds	SD, SC, SE-M, ST	N	3
Fabaceae	*Neltuma ruscifolia* (Griseb.) C.E. Hughes & G.P. Lewis	viñal	tree	dried leaves	SE-M, ST, SD	N	3
Fabaceae	*Peltophorum dubium* (Spreng.) Taub.	yvyrá pytá	tree	Bark	SR, ST, SD	N	7
Fabaceae	*Rhynchosia edulis* Griseb.	urusu he’e	perennial herb	Root	SR, SD	N	6
Fabaceae	*Senna alexandrina* Mill.	sen	shrub	leaves	SD	I	2
Fabaceae	*Senna occidentalis* (L.) Link	taperyva hú, eupatorio falso, taperyrá	suffrutescent herb	root, bark, leaves, seeds	ST, SD, SC, SR, SG-U	N	6
Fabaceae	*Vachellia farnesiana* (L.) Wight & Arn. Willd.	aromita	tree	flower, leaves and fresh plant, fruits	SD, SC, SN, ST	N	4
Fabaceae	*Vachellia aroma* (Gillies ex Hook. & Arn.) Seigler & Ebinger	aromita, tusca	tree	flower, leaves, aloja (fermented beverage)	SD, SC, SE-M, SG-U, ST	N	3
**Gentianaceae**	***Gentianella alborosea* (Gilg) Fabris**	**hercampure**	**herb**	**Bark**	**SD, SC, SE-M**	**I**	**0**
Hypericaceae	*Hypericum connatum* Lam. *	ka’avo tory	herb	flowering tops	SN, SD, SC, SE-M	N	2
Iridaceae	*Trimezia spathata* (Klatt) Baker	ruibarbo	herb	fresh rhizome	SD, SG-U	N	5
Lamiaceae	*Hyptis brevipes* Poit.	kavará ka’a, yerba de lucero	annual herb	aerial part	SE-M, SD	N	3
Lamiaceae	*Lavandula angustifolia* Mill.	alhucema, lavándula	suffrutescent herb	aerial part	SE-M, SG-U, SD, SN, ST	I	3
Lamiaceae	*Leonotis nepetifolia* (L.) R. Br.	cordón de fraile	herb	leaves	SC, SE-M, SM-E	N	3
Lamiaceae	*Melissa officinalis* L.	melisa, toronjil	herb	leaves, flowering top	SN, SM-E, SD, ST, SR	I	5
Lamiaceae	*Mentha piperita* L.	menta’i, hierba mota	herb	fresh plant	SD, SM-E	I	7
Lamiaceae	*Mentha spicata* L. var. *rotundifolia* L.	hierba buena	herb	leaves	SD	I	4
Lamiaceae	*Ocimum basilicum* L.	albahaca	herb	leaves	SD, SN, ST	I	4
Lamiaceae	*Ocimum ovatum* Benth.	ka’a katí, ka’i ka’a	suffrutescent herb	leaves	SC, SN	N	3
Lamiaceae	*Origanum vulgare* L.	orégano	herb	leaves and flowering tops	SD, SE-M, SR, SG-U	I	3
Lamiaceae	*Salvia rosmarinus* Spenn.	romero	shrub	leaves, flowers	ST, SD, SC, SG-U	I	7
Lamiaceae	*Vitex megapotamica* (Spreng.) Moldenke	tarumá	tree/shrub	root, branches, leaves	SC, SD, SG-U	N	1
Lauraceae	*Cinnamomum verum* J.Presl	canela	tree	bark, leaves	SD, SG-U, SN	I	4
Lauraceae	*Laurus nobilis* L.	laurel de España	tree	leaves	SD, SE-M, SM-E	I	3
Lauraceae	*Nectandra angustifolia* (Schrad.) Nees & Mart.	ajuý	tree	leaves, fruits	SM-E, SE-M, SD, SC, SG-U	N	3
Lauraceae	*Persea americana* Mill.	aguacate, apeterevy	tree	leaves and buds, bark	SC, SD, SE-M, SM-E	I	6
Linaceae	*Linum usitatissimum* L.	lino	herb	seeds, seed powder	SD, SR	I	4
Loranthaceae	*Psittacanthus divaricatus* (Kunth) G. Don	ka’avo tyry’y	hemiparasitic herb	leaves, young stems, flowers	SC, SN, SD	N	2
Lycopodiaceae	*Lycopodiella cernua* (L.) Pic.Serm.	itá poty	herb	spores	ST, SG-U, SD	N	3
Lythraceae	*Cuphea lysimachioides* Cham. & Schltdl.	ysypo pere	herb	whole plant, rhizome and root	SD, SR, SE-M, ST	N	5
Lythraceae	*Cuphea racemosa* (L.f.) Spreng.	siete sangría	herb	flowering plant	SD, SC, SE-M, SM-E	N	7
Lythraceae	*Heimia salicifolia* Link	yerba de la vida	suffrutescent herb	aerial part, leaves, root	SC, SD, ST	N	4
Lythraceae	*Punica granatum* L.	granada	tree	dried fruit husk	SD, SG-U	I	5
Malpighiaceae	*Heteropterys glabra* Hook. & Arn.	tilo falso	scrambling shrub	flowering top, leaves and fruits	SC, SN	N	6
Malpighiaceae	*Heteropterys brachiata* (L.) DC.	nudo cachorro, ñudo cachorro	shrub	root	SC, SD, SM-E, SG-U	I	2
Malvaceae	*Ceiba chodatii* (Hassl.) Ravenna *	palo borracho, yuchan, samu’u, cucó	tree	prickles, thorns	SG-U, SM-E, SE-M, SD, ST	N	3
Malvaceae	*Ceiba speciosa* (A. St.-Hil.) Ravenna *	samu’u	tree	thorns and bark	ST, SE-M, SG-U	N	1
Malvaceae	*Cienfuegosia drummondii* (A. Gray) Lewton	guaikuru ka’a	herb	whole plant	SG-U, ST	N	3
Malvaceae	*Guazuma ulmifolia* Lam.	kambá aká	tree	bark	SD, SE-M	N	2
Malvaceae	*Hibiscus sabdariffa* L.	flor de jamaica, rosa piola, rosella	suffrutescent herb	calyx	SD	I	1
Malvaceae	*Luehea divaricata* Mart.	ka’a ovetí, azota caballo	tree	flower, leaves, bark	SR, SE-M, SD, ST, SM-E	N	6
Malvaceae	*Sida cordifolia* L.	malva blanca	perennial herb	flowering top, root	SR, SD, ST, SG-U, otros	N	12
Malvaceae	*Sida spinosa* L.	typychá hu	annual herb	aerial part	SD, SG-U, SE-M, SN	N	4
Malvaceae	*Sidastrum paniculatum* (L.) Fryxell	makagua ka’a	herb	leaves	SD, SG-U, ST	N	3
Meliaceae	*Cedrela fissilis* Vell. *	cedro	tree	bark, fruits	SE-M, ST	N	2
Meliaceae	*Melia azedarach* L.	paraiso	tree	leaves, branches, fruits	ST	I	4
Menispermaceae	*Cissampelos pareira* L.	mil hombre’i, avutua, ka’apeva, jaguareté nambí	liana	stem, root, leaves	SN, SE-M, SC, SD, ST, SG-U,	N	3
Monimiaceae	*Peumus boldus* Molina	boldo	shrub	leaves	SD, SN, SC, SE-M	I	3
Moraceae	*Dorstenia brasiliensis* Lam.	tarope, contrayerba, ka’apiá	perennial herb	whole plant, leaves	SC, SM-E, SG-U	N	11
Moraceae	*Ficus carica* L.	higuera	tree	leaves	SE-M	I	3
Moraceae	*Maclura tinctoria* (L.) D. Don ex Steud. subsp. *tinctoria*	tata jyva	tree	dried bark, fruit, latex	SD, SG-U, SN, SE-M, ST	N	3
Moraceae	*Morus alba* L.	mora	tree	leaf	SE-M, SC	I	4
Moraceae	*Sorocea bonplandii* (Baill.) W.C. Burger, Lanj. & Wess. Boer	ñandypami, kangorosa guasú	tree	bark	SG-U, SM-E	N	3
Moringaceae	*Moringa oleifera* Lam.	moringa	tree	leaf	SE-M	I	2
Myrtaceae	*Campomanesia pubescens* (DC.) O. Berg	guavirami	shrub	fruit, leaves	SD	N	5
Myrtaceae	*Campomanesia xanthocarpa* (Mart.) O.Berg	guavirá pytá	tree	dried leaves, fruit	SD, SC, ST	N	3
Myrtaceae	*Corymbia citriodora* (Hook.) K.D.Hill & L.A.S.Johnson	eucalipto medicinal, mentolado	tree	leaves, fruits	SR	I	1
Myrtaceae	*Eucalyptus* sp.	eucalipto	tree	leaf	SR	I	4
Myrtaceae	*Eugenia myrcianthes* Nied.	yva hái, yva hai mi	tree	leaves	SE-M, SD	N	4
Myrtaceae	*Eugenia uniflora* L. *	ñangapiry	tree	fresh or dried leaves, bark	SC, SE-M, SD, SG-U, ST	N	9
Myrtaceae	*Psidium grandifolium* DC. *	katuáva guasu	shrub	leaves	SD, SG-U, SE-M, SN	N	4
Myrtaceae	*Psidium guajava* L.	guayaba	tree	leaves, root, husk, dried fruits	SD, SG-U	I	9
Nyctaginaceae	*Boerhavia diffusa* L.	ka’arurupe, tangará, anleewa	perennial herb	root	SC, SD	N	7
Nymphaeaceae	*Victoria cruziana* Orb. *	jakaré yrupé, maíz de agua	aquatic-marsh herb	flowers, seeds	SD, SN, SG-U	N	3
Orchidaceae	*Cohniella jonesiana* (Rchb. f.) Christenson *	suelda consuelda	epiphytic herb	whole plant	SG-U	N	1
Orchidaceae	*Cyrtopodium palmifrons* Rchb.f. & Warm.	tamanakuna	epiphytic herb	stems	SE-M, SM-E	N	1
Orobanchaceae	*Escobedia grandiflora* (L. f.) Kuntze *	ysypó ju, azafrán del pais, azafranillo	suffrutescent herb	root	ST, SE-M, SM-E	N	1
Papaveraceae	*Argemone subfusiformis* G.B. Ownbey	cardo santo	herb	roots and seeds	SG-U, SC, ST, SN, SD	Nat	8
Passifloraceae	*Passiflora cincinnata* Mast.	mburukujá pytá, mburukuja’i	climbing herb	leaves	SC, SN	N	5
Petiveriaceae	*Petiveria alliacea* L.	pipi, lemtewei	annual herb	leaves, whole plant, root	SC, ST, SG-U, SM-E, SR, SD	N	8
Phyllanthaceae	*Phyllanthus niruri* L.	para para’i	herb	aerial part	SG-U	N	5
Phyllanthaceae	*Phyllanthus orbiculatus* Rich.	para para’i	annual herb	whole plant	SD, SG-U, SC, SN	N	3
Phyllanthaceae	*Phyllanthus sellowianus* (Klotzsch) Müll. Arg.	sarandí blanco	shrub	wood, root, leaves	SC, SD, SE-M, SG-U	N	3
Piperaceae	*Peperomia circinnata* Link	jatevu ka’a	epiphytic herb	whole plant	SD, SC, SN, SG-U	N	5
Piperaceae	*Peperomia tetraphylla* (G. Forst.) Hook. & Arn. *	ypeku ka’a	epiphytic herb	fresh plant	SD, SC	N	2
Piperaceae	*Piper regnellii* (Miq.) C. DC. *	jaguarundi	shrub	fresh leaves	SR, SD	N	12
Plantaginaceae	*Plantago tomentosa* Lam.	llantén kokue, ka’a juky	perennial herb	whole plant	SM-E, SD, SC, ST, SR	N	10
Plantaginaceae	*Scoparia dulcis* L.	typychá kuratú, escobilla	herb/shrub	aerial part, whole plant with root	SR, SG-U, SD, ST	N	7
Poaceae	*Arundo donax* L.	caña de castilla	herb	rhizome	SC, SD, SE-M	N	3
Poaceae	*Cymbopogon citratus* (DC.) Stapf	cedrón kapi’i	herb	aerial part	SD	I	10
Poaceae	*Digitaria horizontalis* Willd.	cebadilla pe	herb	vegetative parts	SE-M, SG-U	N	3
Poaceae	*Digitaria insularis* (L.) Mez ex Ekman	kapi’i pororo	perennial herb	aerial part	SN	N	3
Poaceae	*Elionurus muticus* (Spreng.) Kuntze	espartillo guasú	herb	dried plant	SC, SD, SN	N	7
Poaceae	*Hordeum vulgare* L.	cebada	herb	fruits	SD, SC, ST	I	3
Poaceae	*Imperata brasiliensis* Trin.	jahape	herb	dried flowers and rhizome	SC, SE-M, SG-U	N	3
Poaceae	*Lithachne pauciflora* (Sw.) P. Beauv.	ka’i arro	perennial herb	aerial part	SE-M	N	1
Poaceae	*Paspalum vaginatum* Sw.	gramilla	herb	roots	SC, SD	N	4
Poaceae	*Zea mays* L. subsp. *Mays*	avatí, avatí ragüe, maíz	herb	styles (silk of mature flowers)	SC, SG-U, SD	I	5
Polygonaceae	*Muehlenbeckia sagittifolia* (Ortega) Meisn.	zarzamora, juapeká rä	vine	stems and roots	SG-U, SC, SD, ST	N	3
Polygonaceae	*Persicaria punctata* (Elliott) Small	ka’a tái, hiedra acre, hierba de bicho	perennial herb	fresh leaves	SG-U, ST, SD	N	7
Polygonaceae	*Salta triflora* (Griseb.) Adr. Sanchez	duraznillo, guaigui piré, ecac, filek (maká)	tree	bark, leaves, root	SD, SR, SE-M, SN	N	3
Polypodiaceae	*Campyloneurum phyllitidis* (L.) Presl.	kalagua’i	herb	whole plant	SG-U, SE-M, SC, SD, SR, SN	N	5
Polypodiaceae	*Microgramma vacciniifolia* (Langsd & Fisch) Copel	angujá ruguai	climbing herb	whole plant	SD, SC, SG-U, ST	N	6
Pontederiaceae	*Pontederia crassipes* Mart.	aguapé	aquatic-marsh herb	flowers and leaves.	ST, SC, SN	N	4
Portulacaceae	*Portulaca oleracea* L.	ka’a pungá, verdolaga	herb	leaves	SD, SC	N	4
Pteridaceae	*Adiantopsis chlorophylla* (Sw.) Fée *	doradilla crespa	herb	whole plant	SG-U, SD, SE-M	N	3
Pteridaceae	*Adiantum pseudotinctum* Hieron.	culantrillo ka’aguy, culantrillo arroyo	fern	plant alone or combined with others	SE-M, SC, SG-U, SR,	N	5
Pteridaceae	*Adiantum raddianum* C. Presl	culantrillo de pozo	fern	aerial part	SD, SG-U, SE-M	N	3
Pteridaceae	*Hemionitis rufa* (L.) Sw. *	doradilla	herb	whole plant	SG-U, SC, SR, SE-M	N	4
Rhamnaceae	*Rhamnidium elaeocarpum* Reissek	tarumá’i	tree	leaves	SG-U, ST, SE-M, SD, SC, SM-E	N	2
Rhamnaceae	*Rhamnus cathartica* L.	cáscara sagrada	tree	bark	SD	I	1
Rosaceae	*Eriobotrya japonica* (Thunb.) Lindl.	nispero	tree	leaf	SD, SE-M	N	3
Rosaceae	*Rosa banksiae* W.T. Aiton	rosa mosqueta	shrub	flowers	SD	I	6
Rubiaceae	*Genipa americana* L.	ñandypa	tree	leaves, bark	SE-M, SC, SD, SM-E	N	8
Rubiaceae	*Spermacoce verticillata* L.	typychá corredor, typycha aka votó	herb	whole plant	SD	N	3
Rutaceae	*Allophylus edulis* (A. St.-Hil., A. Juss. & Cambess.) Hieron. ex Niederl.	kokú	tree	leaves	SD, SE-M, SM-E	N	9
Rutaceae	*Balfourodendron riedelianum* (Engl.) Engl. *	guatambú	tree	bark	ST	N	1
Rutaceae	*Citrus × aurantium f. aurantium*	apepú hai, azahar	tree	leaves, flowers, fruits, husk	SN, ST	Nat	9
Rutaceae	*Citrus medica* L.	cidra, limón	tree	leaves, seeds, fruit	SD, SN, SR	I	4
Rutaceae	*Citrus sinensis* (L.) Osbeck	naranja dulce	tree	fruit epicarp	SD	I	3
Rutaceae	*Helietta apiculata* Benth.	yvyrá ovi	tree	bark	SD, SE-M, SC	N	3
Rutaceae	*Pilocarpus pennatifolius* Lem.	yvyrá tai, jaguarundí	tree	leaves	ST, SR, SC, SM-E, SE-M	N	7
Rutaceae	*Ruta graveolens* L.	ruda	shrub	leaves	SD, SG-U, SM-E	I	6
Rutaceae	*Zanthoxylum caribaeum* subsp. *rugosum* (A. St.-Hil. & Tul.) Reynel	tembetary hü	tree	leaves	SN, SE-M, SC, SD	N	3
Salicaceae	*Salix humboldtiana* Willd.	sauce criollo	tree	leaves, bark	ST, SE-M, SG-U	N	4
Santalaceae	*Casearia sylvestris* Sw.	burro ka’a, mburiká ka’a	tree	roots and leaves	SD, SM-E, SE-M, SG-U, ST	N	6
Santalaceae	*Phoradendron argentinum* Urb.	ka’avo tyre’y	hemiparasitic herb	whole plant	SG-U, SD	N	2
Santalaceae	*Phoradendron bathyoryctum* Eichler	muérdago, ka’avo tyre’y	hemiparasitic herb	leaves	SG-U	N	2
Santalaceae	*Phoradendron liga* (Gillies ex Hook. & Arn.) Eichler	ka’avo tyrey	hemiparasitic herb	aerial part	SG-U	N	3
Scrophulariaceae	*Buddleja stachyoides* Cham. & Schltdl.	barbasco, mbuirá ka’a, verbasco	subshrub	flowers, leaves	SR, SD, ST	N	9
Simaroubaceae	*Picrasma crenata* (Vell.) Engl. *	para’y, pyre’ro	tree	bark, wood (sawdust), root, leaves	SE-M, SG-U, ST	N	2
Smilacaceae	*Smilax campestris* Griseb.	ju’a peká, turuví	climbing herb	root	SM-E, SG-U, SC, SD, SE-M	N	6
Solanaceae	*Nicotiana tabacum* L.	tabaco, pety	shrub	leaves	SN, ST, SD, SE-M, SM-E, SG-U	I	3
Solanaceae	*Physalis viscosa* L.	kamambu’i	perennial herb	aerial part	SC, SR, SD, SE-M, ST	N	3
Solanaceae	*Solanum americanum* Mill.	arachichú	herb	fresh plant, with fruits	SD, SG-U	N	7
Solanaceae	*Solanum granuloso-leprosum* Dunal	hu’i moneha	tree	root, leaves, fruits	SD, SN, SE-M	N	4
Solanaceae	*Solanum paniculatum* L.	juruvéva	shrub	leaves	SD, SG-U, SE-M, SN, SC	N	3
Solanaceae	*Solanum sisymbriifolium* Lam.	ñuati pyta, mboi rembi’u, revienta caballo, yam meeyak	subshrub	root bark, flowering stems	SC, SD, SM-E, SG-U	N	13
Talinaceae	*Talinum paniculatum* (Jacq.) Gaertn.	verdolaga guasu, paskakma yaamit	perennial herb	leaves	ST, SE-M, SD	N	4
Urticaceae	*Cecropia pachystachya* Trécul	amba’y	tree	leaves, roots, flowering tops, flower buds	SR, SC, SD, ST	N	11
Urticaceae	*Parietaria debilis* G. Forst.	ka’a piky, hierba de San Pedro	annual herb	whole plant	SD, SC, SG-U, SE-M, ST	N	4
Urticaceae	*Urera aurantiaca* Wedd.	pyno pytá, ka’a pochy	shrub	leaves	SE-M, ST, SD, SC	N	3
Urticaceae	*Urera baccifera* (L.) Guadich. Ex Wedd.	pyno guasu	shrub/tree	fresh or dried root, whole plant	SE-M, SC, ST, SD, SM-E, SG-U	N	7
Urticaceae	*Urtica spathulata* Sm.	pyno’i	herb	aerial part	SC, SD, SE-M, SM-E	N	5
Verbenaceae	*Aloysia citrodora* Palau	cedrón Paraguay	shrub	flowering top,	SD, SM-E, SN, SC	I	7
Verbenaceae	*Aloysia gratissima* (Gill. & Hook) Troncoso	poleo’i	shrub	dried aerial part	SD, SG-U, SN	N	9
Verbenaceae	*Aloysia polystachya* (Gris.) Mold.	burrito, poleo, te de burro	shrub	flowering top, leaves	SD, SG-U, ST	I	7
Verbenaceae	*Citharexylum myrianthum* Cham.	paloma rembi’ú	tree	bark	SE-M	N	3
Verbenaceae	*Lantana camara* L.	ka’a mara, kamara	shrub	leaves	SD, SR	I	2
Verbenaceae	*Lippia alba* (Mill.) N.E.Br.	salvia, aasik yaamit	shrub	flowering top, dried plant	SG-U, SD, SR	N	7
Verbenaceae	*Lippia brasiliensis* (Link) T. Silva	jate’i ka’a ka’aguy	shrub	leaves	SM-E, SR, SD	N	4
Verbenaceae	*Stachytarpheta cayennensis* (Rich.) Vahl	tatú ruguái, aguará pondá	suffrutescent herb	fresh flowering plant	SD, SN, SC, SG-U	N	5
Verbenaceae	*Verbena litoralis* Kunth	verbena’i	perennial or biennial herb	aerial part	SR, SD, SC	N	6
Viburnaceae	*Sambucus australis* Cham. & Schltdl.	sauco, akapó	tree/shrub	leaves, bark, flowers	SD, SR, SM-E, SC, ST, SE-M	I	5
Vitaceae	*Cissus verticillata* (L.) Nicolson & C.E.Jarvis	insulina	climbing herb	stems, leaves and roots	SR, SE-M	N	3
Vitaceae	*Vitis vinifera* L.	vid	vine	leaves	ST, SE-M, SD	I	2
Zingiberaceae	*Hedychium coronarium* J. Koenig	flor de caña	herb	rhizome	SN, SR	I	4
Zingiberaceae	*Zingiber officinale* Roscoe	gengibre	herb	rhizome	SD, SE-M	I	2
Zygophyllaceae	*Plectrocarpa sarmientoi* (Lorentz ex Griseb.) Christenh. & Byng *	palo santo, guayacán, yvyrá okái	árbol	corteza, madera (aserrin)	ST, SD, SC, SR, SM-E, SG-U,	N	8

**Figure 2:**
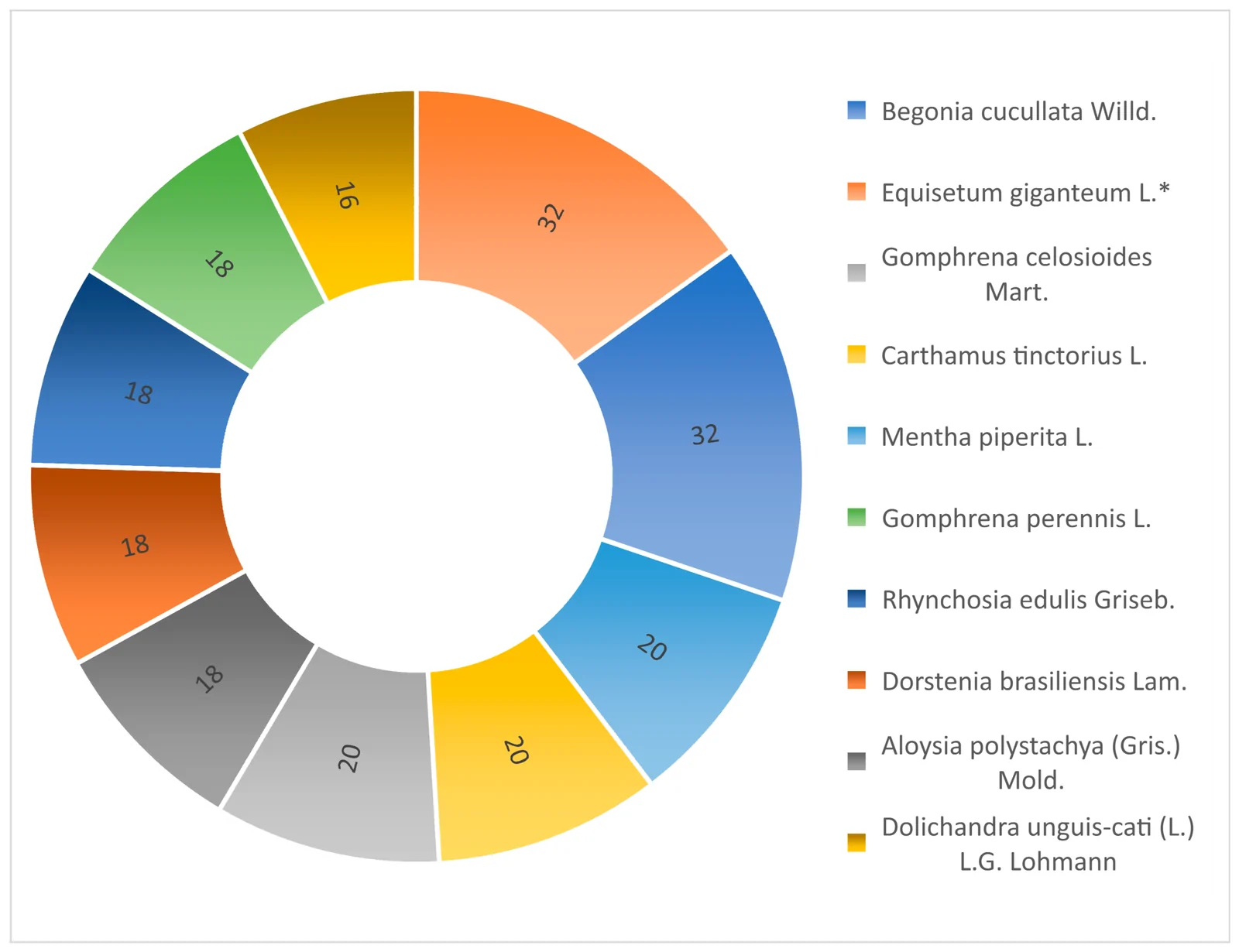
Frequency of mention of species in the interviews. * The graph shows the relative proportion of the ten most frequently cited plant species.

The graph shows the relative proportion of the ten most frequently cited plant species. *Equisetum giganteum* L. and *Begonia cucullata* Willd. are represented more frequently, while the other species have lower and relatively similar proportions. *Dolichandra unguis-cati* (L.) L.G. Lohmann is the least frequently cited species. The graph does not include the remaining 130 species that were mentioned by the contributors between 1 and 8 times (Figure 2).

A significant proportion of the flora recorded in Paraguay (16%) has medicinal uses.

### 2.3. Officinal Part Used in Traditional Medicine

**Figure 3:**
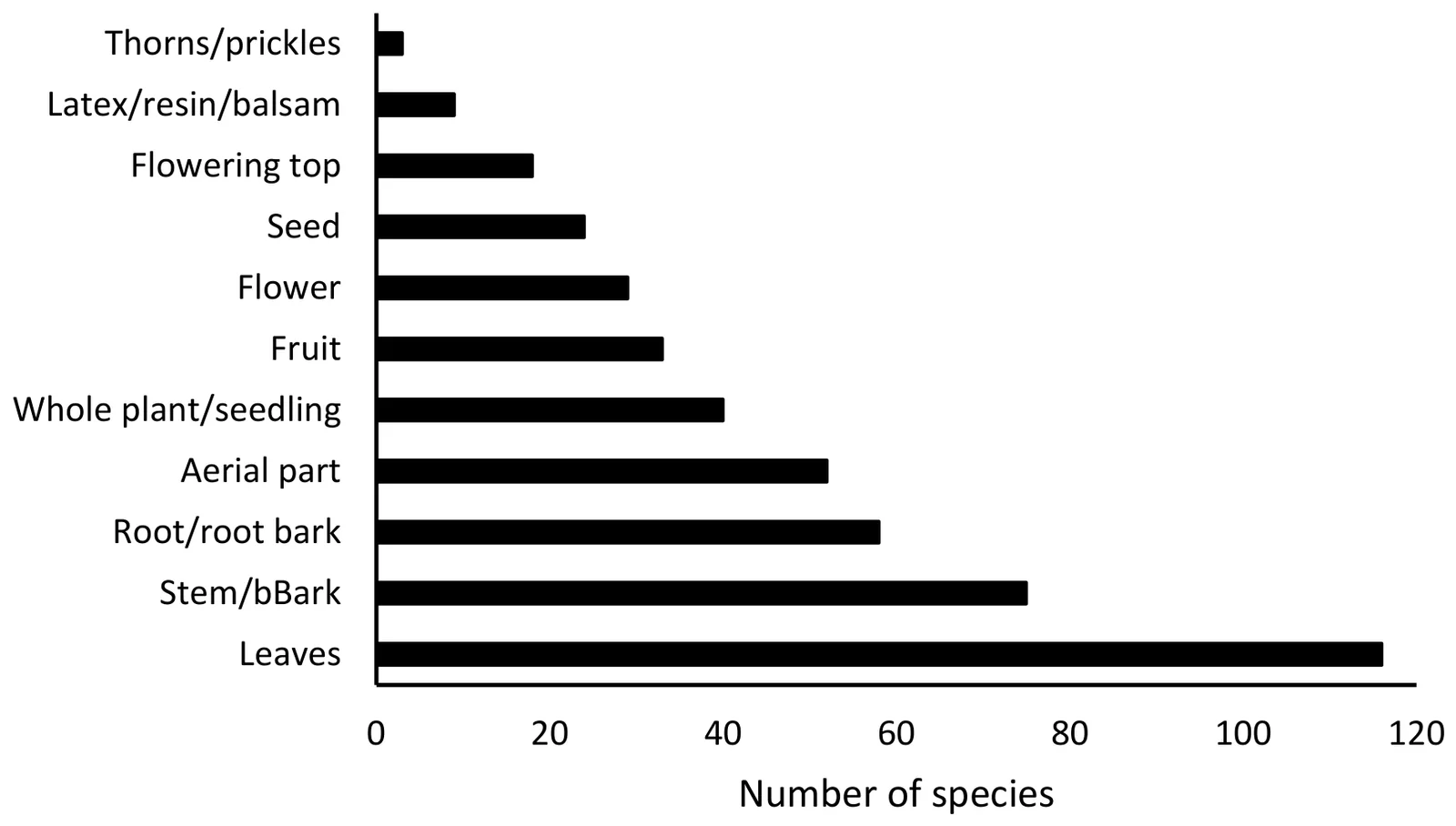
Number of medicinal species recorded according to the officinal part used.

With regard to the officinal part utilised, it has been documented that leaves are employed in the majority of species, followed by stems/bark, roots/bark, aerial parts, entire plants, fruits, flowers, seeds, flowering tops, latex/resin/balsam, and thorns/prickles (Figure 3). The result obtained in this study is consistent with those reported in studies conducted in Colombia [15] and Ecuador [16,18].

The primary example of this is yerba mate leaves, which are utilised in the infusion of the same name, a beverage that is highly popular in Argentina, Uruguay, Paraguay and southern Brazil [20], with Paraguay exhibiting the most extensive acceptance of this medicinal plant.

The consumption of *mate* and *tereré* is a daily occurrence. Furthermore, it is important to note that they fulfil a distinctive social function. It is a common occurrence for multiple individuals to share a single *mate*, *guampa* and *bombilla* [35]. In addition, these beverages represent a significant source of caffeine intake [20].

### 2.4. Body Systems Targeted by Medicinal Species

**Figure 4:**
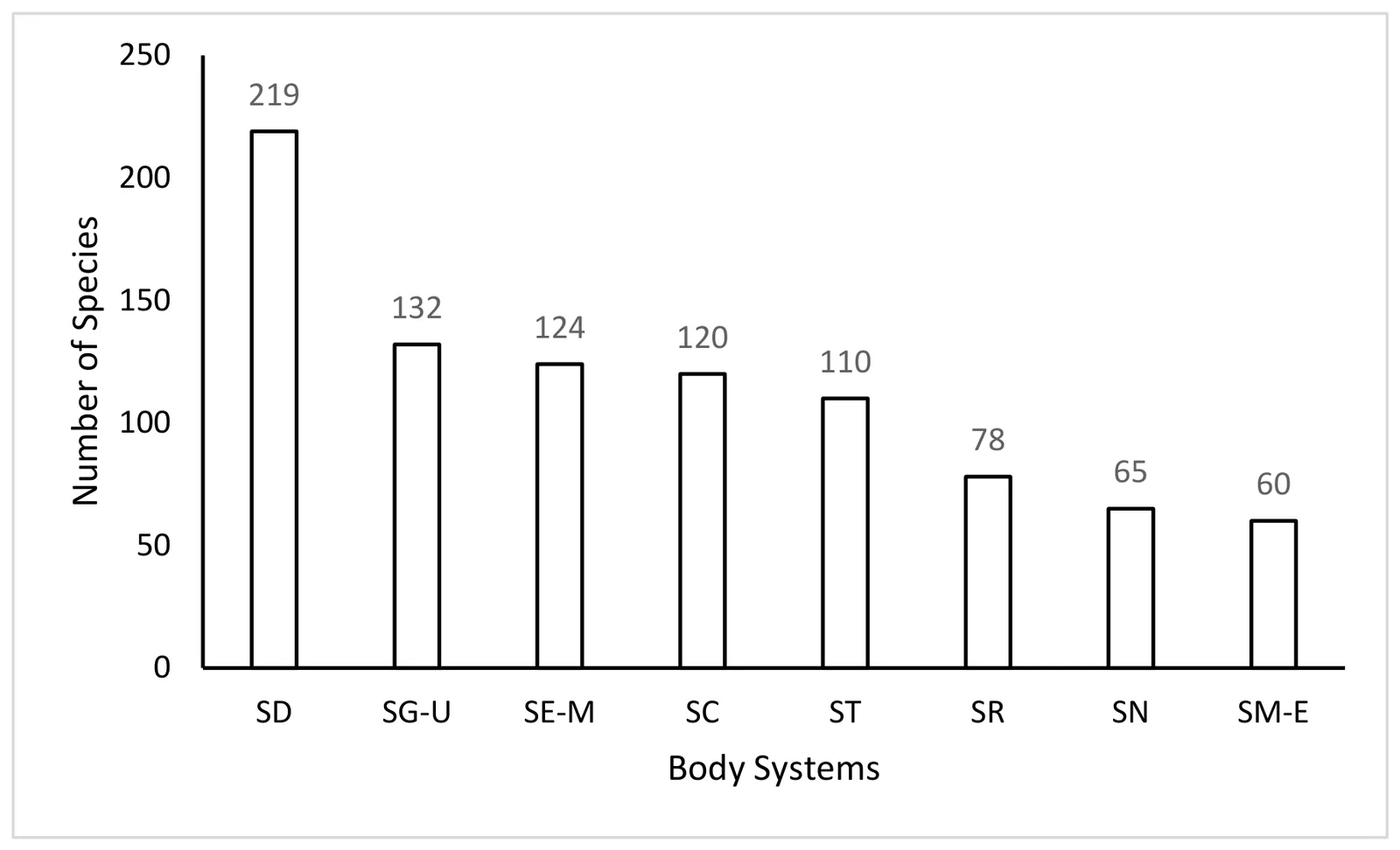
Number of species acting on the body systems.

The majority of the documented species exhibit their effects on the digestive system, with the next most prevalent categories being the genitourinary, endocrine–metabolic, circulatory, integumentary, respiratory, nervous and musculoskeletal systems (Figure 4). The present result is consistent with the findings of studies conducted in Mexico [14], Colombia [15], Ecuador [16] and Peru [13,40], which recorded a significantly higher prevalence of plant utilisation for the treatment of digestive ailments.

The addition of medicinal plants to *mate* and *tereré* is intended to enhance flavour, but these plants are also utilised by the local population to alleviate symptoms of ailments such as headaches and gastrointestinal disorders [41,42]. Examples of such species include the *agrial* (*Begonia cucullata*), the *kangorosa* (*Maytenus ilicifolia*), and the *jaguá rundi* (*Piper regnellii*). Whilst there is a general tendency for conventional remedies to be substituted with pharmaceuticals, particularly in urban areas, ethnobotanical and epidemiological studies demonstrate the continued relevance of ethnopharmacological knowledge with respect to the prevention and treatment of various ailments [19,43].

### 2.5. Medicinal Species Included in a Category of MADES Protection

Threats to biodiversity are increasing on a global scale, and some medicinal plants are subject to some form of protection. At the national level, two resolutions—512/25 from the Ministry of Environment and Sustainable Development (MADES) [12] and 1609/06 from the Secretariat of the Environment of Paraguay—establish lists of threatened species [39]. The present study documented 33 medicinal species that were included in the aforementioned list, which were classified within one of several threat categories. Of these, 18 are classified as Endangered (EN), 8 as Vulnerable (VU), and 7 as Threatened with Extinction (TE) (Table 2).

In line with the interviews and reviews conducted, of the 33 threatened species, 17 were recorded in the references, and 16 were reported in the interviews carried out in this study. The species in question are *Piper regnellii* (EN), *Baccharis trimera* (VU), *Maytenus ilicifolia* (EN), *Herreria montevidensis* (EN), *Achyrocline alata* (VU), *Stevia rebaudiana* (EN), *Eugenia uniflora* (VU), *Equisetum giganteum* (EN), *Plectrocarpa sarmientoi* (EN), *Libidibia paraguariensis* (EN), *Psidium grandifolium* (EN), *Hemionitis rufa* (EN), *Baccharis crispa* (VU), *Cyclolepis genistoides* (VU), *Myrocarpus frondosus* (EN), *Ceiba chodatii* (AE), *Victoria cruziana* (VU), and *Adiantopsis chlorophylla* (EN) (Table 2).

A mere 15 of these plants are included in the IUCN Red List (Table 2) [44]. Notable examples include *Araucaria angustifolia*, categorised as Critically Endangered (CR), *Balfourodendron riedelianum* and *Plectrocarpa sarmientoi*, both classified as Endangered (EN), and *Libidibia paraguariensis*, designated as Vulnerable (VU). In the category of Least Concern (LC), the following species are included: *Annona cacans*, *Apuleia leiocarpa*, *Ceiba speciosa*, *Dimorphandra mollis*, *Equisetum giganteum*, *Eugenia uniflora*, *Handroanthus heptaphyllus*, *Maytenus ilicifolia*, *Psidium grandifolium*, *and Rauvolfia sellowii*. *Myrocarpus frondosus* is categorised as Data Deficient (DD). It is important to recall that the CR and EN threat categories, according to the IUCN (2012) [45], refer to species facing a high risk of extinction in the wild due to population decline or limited geographic distribution.

The protected species *Herreria montevidensis* (a vine whose swollen root is used) and *Maytenus ilicifolia* (a shrub whose leaves and the bark of its roots and stems are utilised) were recorded in all the markets analysed; both are classified as Endangered (EN) by MADES.

**Figure 5:**
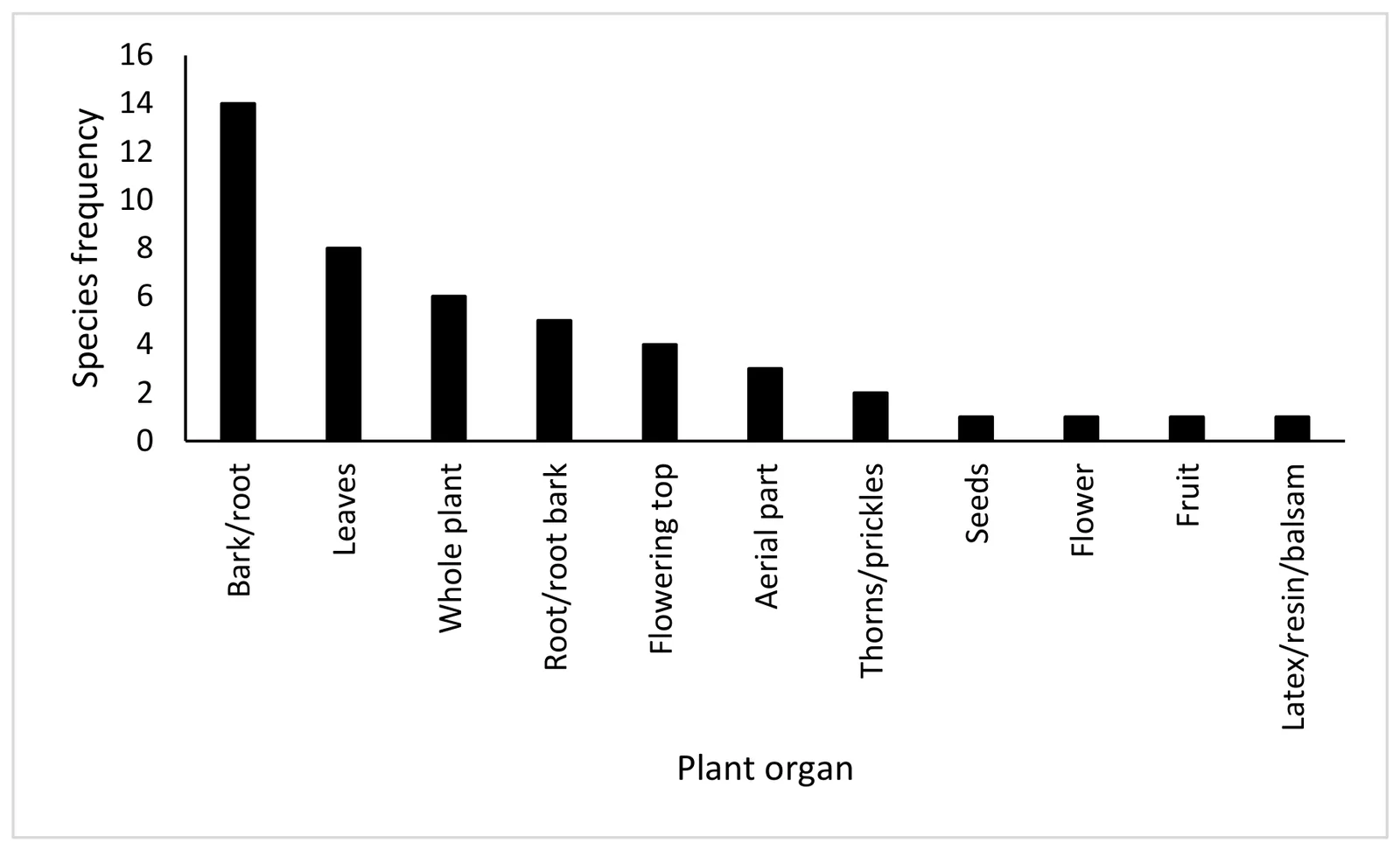
Frequency of species used according to plant organ.

Additionally, traders sold seven species assessed on the IUCN Red List, ranging from the Least Concern (LC)—*Dimorphandra mollis*, *Equisetum giganteum*, *Eugenia uniflora*, *Maytenus ilicifolia* and *Psidium grandifolium*—to species with a higher threat status such as *Libidibia paraguariensis* (VU) and *Plectrocarpa sarmientoi* (EN). In these species, the part used for medicinal purposes is their bark or wood; handlers must therefore manage them with great care and in an appropriate manner. These parts are, moreover, frequently used in plants classified as being under threat (Figure 5).

It should be noted that *Equisetum giganteum*, classified as EN and LC, is one of the species most frequently mentioned in the interviews.

**Figure 6:**
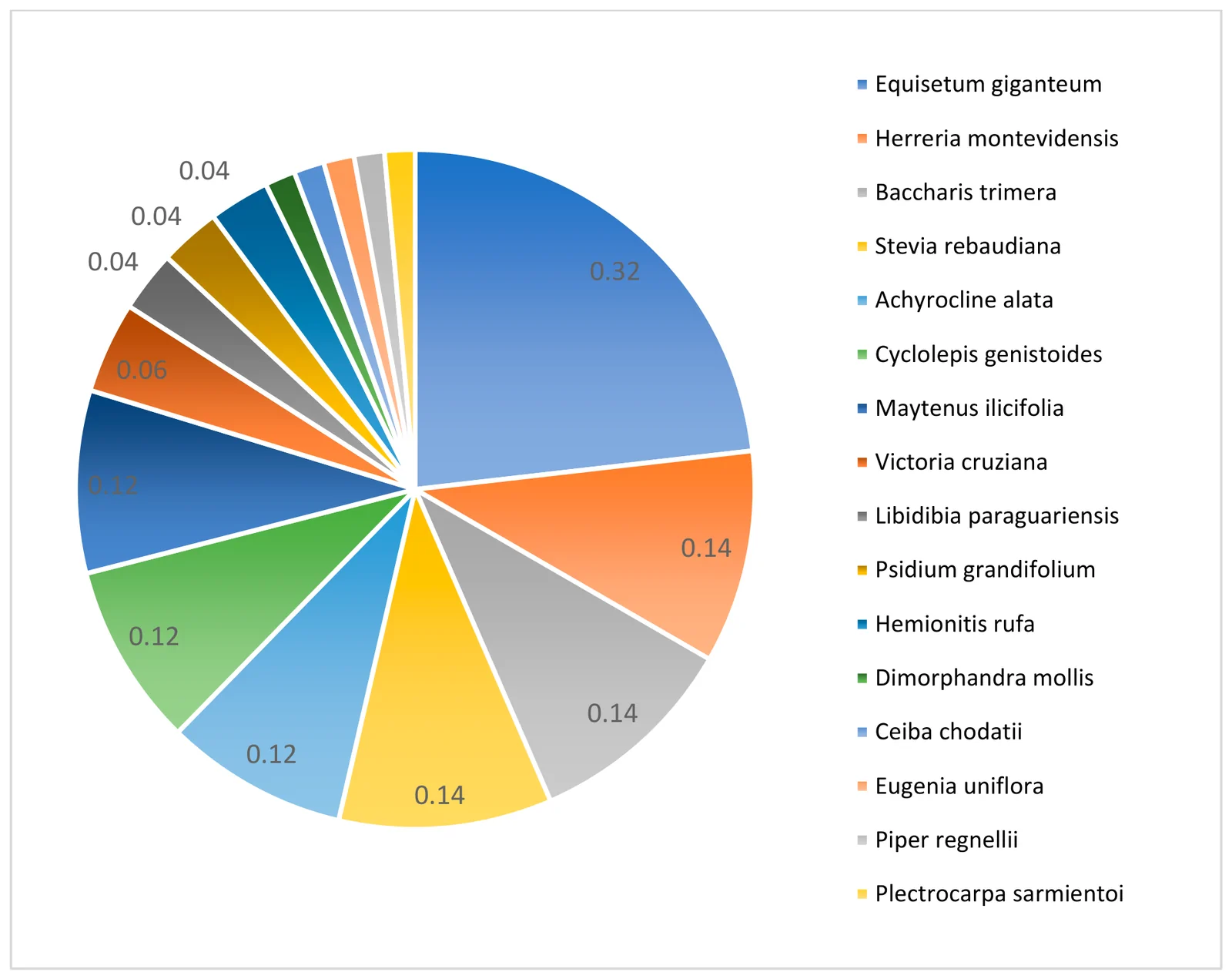
Index of cultural importance of the threatened species mentioned in the interviews.

*Equisetum giganteum* is the most widely recognised species within the traditional knowledge of the studied population. It is followed by *Herreria montevidensis*, *Baccharis trimera*, and *Stevia rebaudiana*, which are also well recognised among the interviewees. On the other hand, *Achyrocline alata*, *Cyclolepis genistoides*, and *Maytenus ilicifolia* show an intermediate index value; finally, *Victoria cruziana*, *Libidibia paraguariensis*, *Psidium grandifolium*, *Hemionitis rufa*, *Dimorphandra mollis*, *Ceiba chodatii*, *Eugenia uniflora*, *Piper regnellii*, and *Plectrocarpa sarmientoi* have an index below 0.10 (Figure 6).

In Paraguay, plants used in everyday life—such as *Maytenus ilicifolia*, *Baccharis trimera*, *Piper regnellii* and *Psidium grandifolium*—are being depleted on a large scale because they are collected directly from their natural habitats rather than being cultivated sustainably [12].

In addition, the native material sold on the market comes from wild collection, except for *Stevia rebaudiana*, a cultivated native species, for which the last recorded wild collection took place in 1986 [46].

We share with Argentina and Brazil the traditional use of certain medicinal species threatened with extinction, such as *Equisetum giganteum*, *Eugenia uniflora*, and *Libidibia paraguariensis*. Medicinal species, as well as traditional knowledge, transcend political borders. The greatest regional threats stem from habitat fragmentation and deforestation [44]. This differs from the situation in Argentina and Brazil, where the commercialisation of herbal medicines relies on cultivation chains, which partially protects wild populations.

Medicinal species have diverse life forms and thrive in different habitats; according to Chaturvedi et al., habitat characteristics—specifically species diversity, functional diversity, tree size, and disturbance indicators—modulate carbon sequestration capacity in tropical dry forest patches [47], which could influence the preservation of medicinal resources, affecting traditional knowledge over time as these resources become scarcer and less accessible.

## 3. Materials and Methods

### 3.1. Study Area

**Figure 7:**
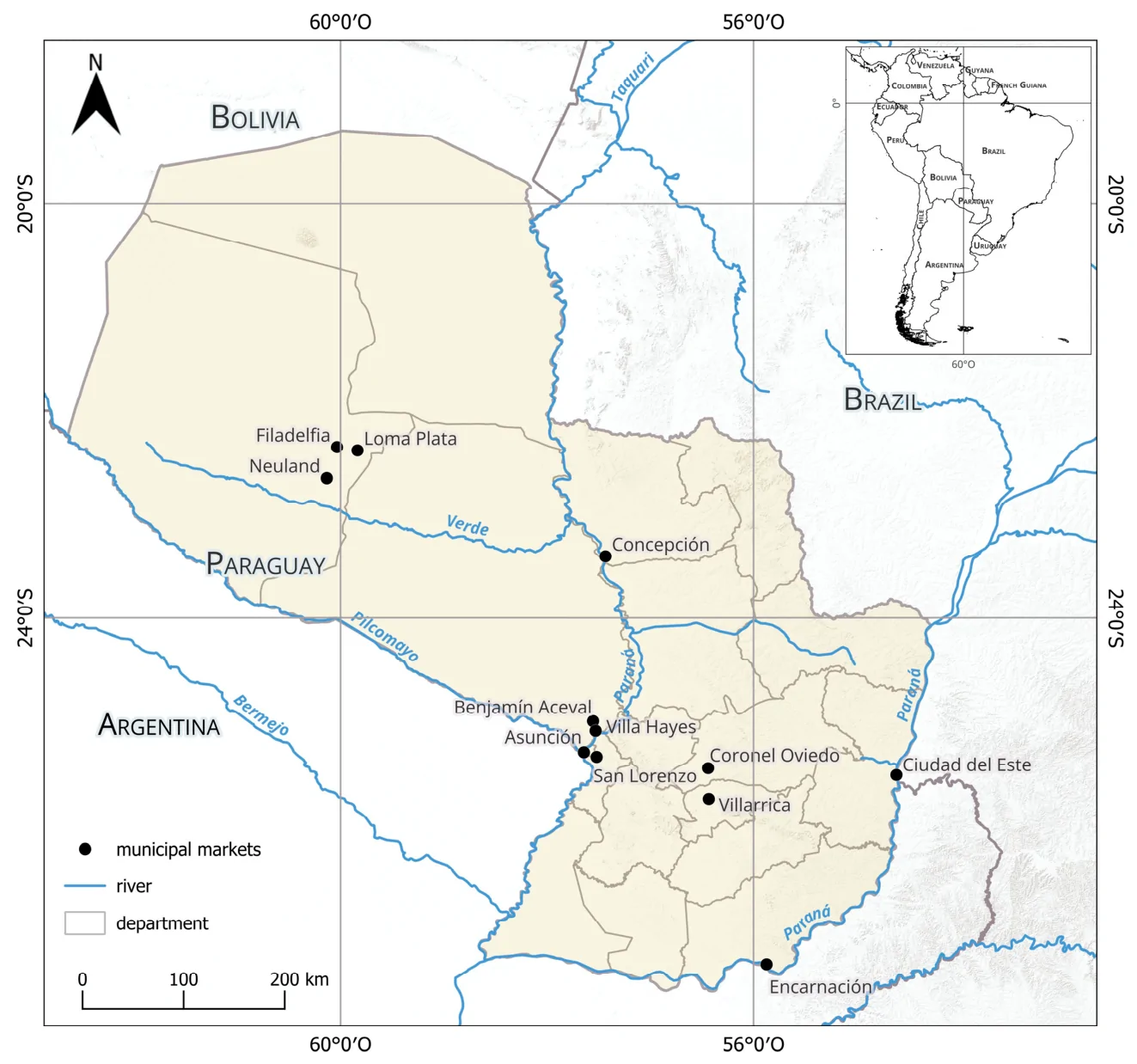
Locations of interview sites in city municipal markets.

The present study was carried out in Paraguay, a subtropical country located in South America, covering 406,752 km^2^ and bordered to the south by Argentina, to the northeast by Brazil, and to the northwest by Bolivia. The country is divided into two distinct natural regions: The Eastern region, which is characterised by a humid, warm climate, and the Western region, otherwise known as Chaco, which is distinguished by a dry, hot climate. These regions are separated by the Paraguay River [48,49] (Figure 7).

### 3.2. Medicinal Uses Prospection (Bibliographic Review)

In accordance with the guidelines of the PRISMA 2020 statement [50] for original systematic reviews, updated systematic reviews, or continuously updated reviews, a search was conducted for publications related to medicinal plants in the ISI Web of Science, Springer, EBSCO, PROQUEST, and Wiley databases. The following keywords were used: ‘medicinal plants’, ‘Paraguay’, ‘traditional knowledge’, ‘ethnobotany’. A combination of keywords was incorporated into the search string using truncation and the Boolean operators AND, OR, quotation marks, the plus sign and asterisks. The following search terms were utilised: “medicinal plants + Paraguay”, “medicinal plants + traditional knowledge”, “medicinal plants + ethnobotany + Paraguay”, “Paraguayan medicinal plants + traditional knowledge”, “use of medicinal plants + Paraguay”, “medicinal plants + conservation + Paraguay”. The same keywords were used with the “and” and “or” operators.

The inclusion criteria considered were references containing information derived from ethnobotanical interviews conducted in Paraguay; references citing the taxonomic identification of the species mentioned in the publications; and publications issued between 1972 and 2024.

**Figure 8:**
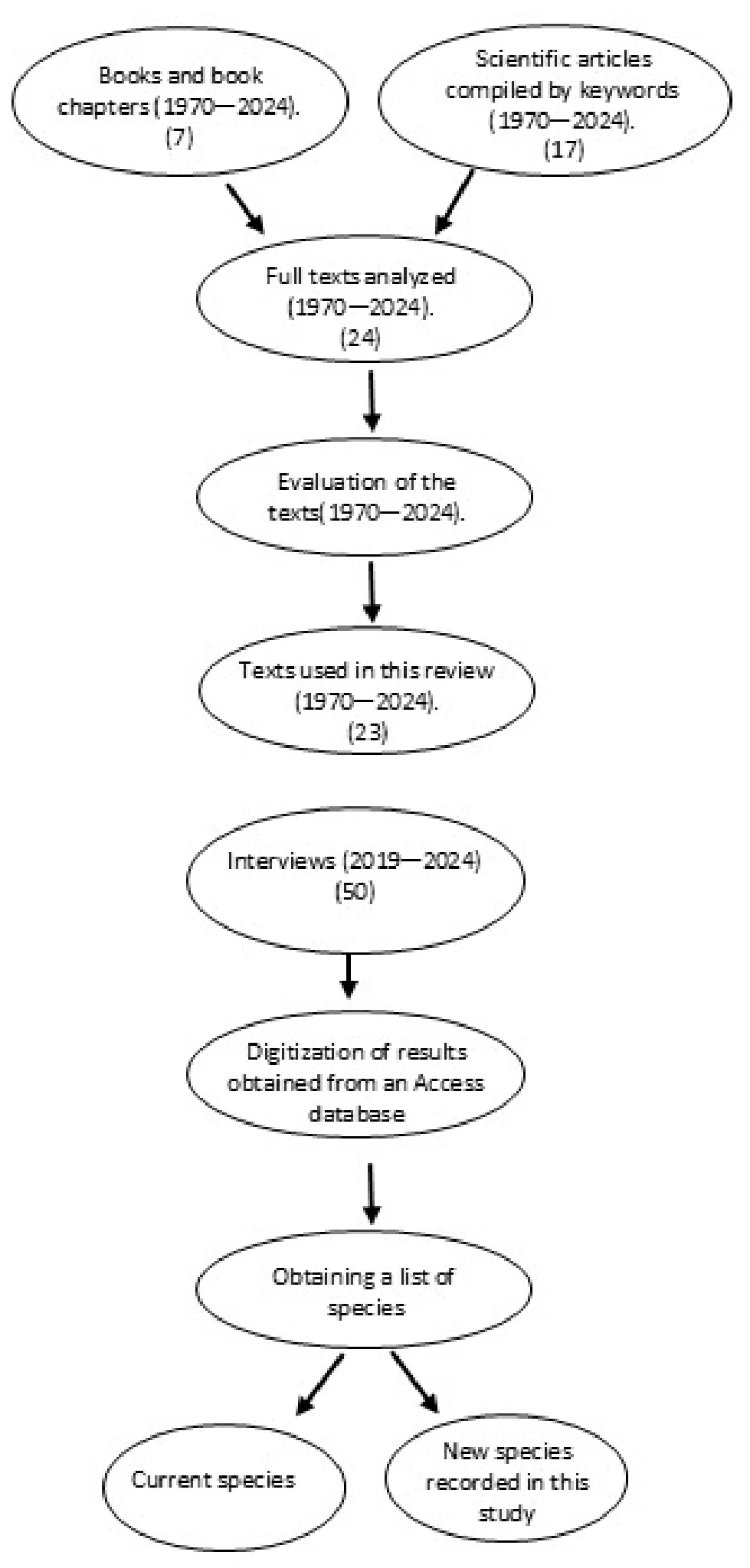
Workflow for the selected information sources, with their quantities listed at the bottom (adapted from [50]), and procedures for processing the interviews.

The initial phase involved removing duplicates, which led to the exclusion of articles, conference abstracts, editorials, and notes. The review considered original articles and books for inclusion. The team compiled a compendium of studies published in English and Spanish. The compilation included a total of 17 scientific articles [43]. Additionally, four complete books provided data not available in the articles (Figure 8).

A comprehensive review of literature was conducted, encompassing books, book chapters, and scientific articles published from 1972 to 2024 [5,6,7,8,9,10,22,23,24,25,26,27,28,29,30,31,32,33,34,35,36,37,38]. Additionally, online databases such as IPNI [51], Darwinion [52], Reflora [53], Trópicos [46], and MPNS [21] were consulted to ensure comprehensive updating of nomenclature and existing synonymies from February to May 2026 (Table 1).

A base list was compiled in a Microsoft Excel spreadsheet containing 863 medicinal species, in which mentions of each species in the various references consulted were recorded in chronological order.

For each species, the following data were recorded: botanical family, genus, scientific name, vernacular name(s), habit, part used, body system affected, native, introduced or naturalised origin, the body system(s) targeted by the species, and the threat category, if any, according to IUCN criteria and AE criteria established by the Ministry of the Environment and Sustainable Development.

The following coding system was employed for the body systems: SC: circulatory system, SD: digestive system, SG-U: genitourinary system, SR: respiratory system, SE-M: endocrine–metabolic system, ST: integumentary system, SN: nervous system, SM-E: musculoskeletal system.

The final list of species was obtained by calculating the frequency of mentions recorded for each species, with a mention being counted as one made by a single author. The selection criterion employed was citation frequency, with a minimum threshold of three mentions in independent references or demonstrated some degree of threat or were subject to legal protection. Furthermore, the national threat category was documented for each species.

### 3.3. Ethnobotanical Survey

A total of 95 semi-structured interviews were conducted with 50 sellers (*yuyeros/as*)/users of medicinal plants [54] in the municipal markets of the following cities: Concepción (Concepción), Ciudad del Este (Alto Paraná), Coronel Oviedo (Caaguazú), Villarrica (Guairá), Encarnación (Itapúa), San Lorenzo (Central), Asunción (Capital), Villa Hayes and Benjamín Aceval (Presidente Hayes), Filadelfia, Neuland and Loma Plata (Boquerón).

The selection of sellers as participants was based on their willingness to engage, with the approach made to stalls situated along the primary access routes of the municipal markets. In each city, 10 sellers were interviewed over the course of one working week, from 7:00 a.m. to 1:30 p.m., coinciding with the stalls’ selling hours. Information was requested from each seller regarding the 10 species deemed most significant to their business. The collaboration between interviewee and interviewer was characterised by its free, voluntary and informed nature, in accordance with the ethical principles outlined in the Faculty of Exact and Natural Sciences (FACEN) ethical code [55]. Key collaborators, selected on the basis of their extensive knowledge of the subject and their greater interest in the project, were interviewed on multiple occasions (Figure 1A–D).

The data presented on the species under investigation were collected between 2020 and 2024. The interviews were recorded, and an informed consent agreement was signed between the interviewee and the interviewer; digital copies of these agreements are stored in the “plantasmedicinalespy” digital folder (“URL: https://drive.google.com/drive/folders/1PINqku-A0y0LFhLwSv0vLT9oTQjSH6VB?usp=drive_link—accessed: 5 October 2026). The medicinal species mentioned were acquired as reference material, identified by specialists, and deposited in the FACEN herbarium [56].

The interviews were conducted to gather information in towns within the departments of Central, Concepción, Guairá, Itapúa, Alto Paraná, Presidente Hayes and Boquerón. The information from the articles, book chapters and books consulted covers the departments of Central, Caaguazú, Caazapá, Canindeyú, Ñeembucú, Boquerón, Presidente Hayes and San Pedro, collectively providing data for 72% of the country’s territory.

### 3.4. Data Analysis

The data obtained from the recorded interviews were digitised and entered into the “Etnobotánica Paraguay” database, which was developed in Microsoft Office Excel.

Subsequent data analysis was conducted by first calculating descriptive statistics. The use of frequencies and percentages was essential for creating statistical tables and graphs, which facilitated the management and processing of the information and the calculation of the cultural importance index for threatened species [57].

## 4. Conclusions

This study compiles a catalogue of 297 species of medicinal plants used in traditional medicine in Paraguay, based on a literature review. Of this total, 139 species were documented through ethnobotanical interviews, including *Gentianella alborosea* (Gilg) Fabris (hercampuri), which previous references for the country had not recorded. The research also documents the medicinal use in Paraguay of 15 species not listed in the Medicinal Plant Names Service (MPNS) database: *Annona cacans*, *Cohniella jonesiana*, *Hemionitis rufa*, *Citharexylum myrianthum*, *Couepia grandiflora*, *Lithachne pauciflora*, *Ocimum ovatum*, *Peperomia circinnata*, *Pereskia nemorosa*, *Rhynchosia edulis*, *Scleria distans*, *Senecio grisebachii*, *Sidastrum paniculatum*, *Trimezia spathata*, and *Viguiera linearifolia*.

In terms of conservation, the study identified 33 medicinal species on the MADES list: 18 Endangered [EN], eight Vulnerable [VU] and seven threatened with extinction [AE], of which only 15 appear on the IUCN Red List. Among the species at greatest risk according to the IUCN, the following stand out: *Araucaria angustifolia* (CR), *Balfourodendron riedelianum* (EN), *Plectrocarpa sarmientoi* (EN) and *Libidibia paraguariensis* (VU). Furthermore, seven species assessed by the IUCN were found to be traded in local markets, ranging from Least Concern (LC)—*Dimorphandra mollis*, *Equisetum giganteum*, *Eugenia uniflora*, *Maytenus ilicifolia* and *Psidium grandifolium*—to species facing a critical level of threat in trade, such as *Libidibia paraguariensis* (VU) and *Plectrocarpa sarmientoi* (EN).

Given the particular vulnerability of *L. paraguariensis* and *P. sarmientoi*, there is a priority to conduct further research into the status of their wild populations and to develop alternatives for sustainable cultivation and propagation. Finally, the study concludes that biocultural conservation in Paraguay requires the participation of local users and traditional gatherers; raising awareness and involving communities is essential not only to safeguard threatened plant resources, but also to preserve the traditional knowledge associated with their use.

## Figures and Tables

**Table 2:** Medicinal species identified in the study that have some form of protection according to the Ministry of Environment and Sustainable Development (MADES, 2025) or the IUCN Red List are detailed. Their growth habit, the part used, the affected body system and the corresponding threat category are specified. **Threat categories**: CR: Critically Endangered, EN: Endangered, VU: Vulnerable, AE: Threatened, LC: Least Concern, DD: Data Deficient.

No.	Family	Species	Vernacular Name	Habit	Part Used	Body System	Threat Category	IUCN Category
1	Annonaceae	*Annona cacans* Warm.	aratikú guasú	tree	seeds, leaves	ST, SR	VU (SEAM 1609/06), EN (MADES 512/25)	LC
2	Apocynaceae	*Rauvolfia sellowii* Müll. Arg.	quina, pseudoquina, palo amargo, kino	tree	bark	SE-M, SD	CR (SEAM 1609/06); EN (MADES 512/25)	LC
3	Araucariaceae	*Araucaria angustifolia* (Bertol.) Steud.	kuri’y	tree	resin (balsam)	ST, SD	EN (MADES 512/25)	CR
4	Asparagaceae	*Herreria montevidensis* Klotzsch ex Griseb.	zarzaparrilla, ysypó mandi’o, pongwanay, jotekatade	vine	root	SD, SG-U, SC, SM-E	EN (SEAM 1609/06), EN (MADES 512/25)	
5	Asteraceae	*Achyrocline alata* (Kunth) DC.	jate’i ka’a	suffrutescent herb	dried plant, leafy-flowering stems	SD, SG-U, SE-M, SM-E, SR	VU (SEAM 1609/06), AE (MADES 512/25)	
6	Asteraceae	*Baccharis crispa* Spreng.	jaguareté ka’a	suffrutescent herb	aerial part	SG-U, SD, SE-M	VU (SEAM 1609/06), AE (MADES 512/25)	
7	Asteraceae	*Baccharis microcephala* (Less.) DC.	jaguareté ka’a, carqueja	subshrub	flowering tops	SG-U, SD	VU (SEAM 1609/06), AE (MADES 512/25)	
8	Asteraceae	*Baccharis trimera* (Less.) DC.	jaguarete ka’a, carqueja	subshrub	aerial part, stems, leaves	SD, SG-U, SM-E, ST	VU (SEAM 1609/06), AE (MADES 512/25)	
9	Asteraceae	*Cyclolepis genistoides* D.Don	palo azul	shrub	aerial part	SD, SM-E, SC, SE-M	VU (SEAM 1609/06), AE (MADES 512/25)	
10	Asteraceae	*Stevia rebaudiana* (Bertoni) Bertoni	ka’a he’é	herb	leaves and dried flowering stems	SE-M, SD, SC	EN (MADES 512/25)	
11	Bignoniaceae	*Handroanthus heptaphyllus* (Vell.) Mattos	tajy hú	tree	bark, flowers	ST, SR	EN (MADES 512/25)	LC
12	Celastraceae	*Maytenus ilicifolia* Mart. ex Reissek	kangorosa, sombra de toro	shrub	branches, fresh leaves, root, whole plant	ST, SD, SG-U, SC, SR	EN (SEAM 1609/06). EN (MADES 512/25)	LC
13	Equisetaceae	*Equisetum giganteum* L.	cola de caballo, kavajú ruguái	herb	sterile stems	SC, SR, ST, SG-U	EN (SEAM 1609/06). EN (MADES 512/25)	LC
14	Fabaceae	*Apuleia leiocarpa* (Vogel) J.F. Macbr.	yvyrá peré	tree	bark	SD	AE (MADES 512/25)	LC
15	Fabaceae	*Dimorphandra mollis* Benth.	barbatimón, lorito pysa	tree	root	SD	EN (SEAM 1609/06), EN (MADES 512/25)	LC
16	Fabaceae	*Libidibia paraguariensis* (D. Parodi) G.P. Lewis	guayacán, carujnangue	tree	bark	SM-E, SG-U, SE-M, SD	AE (MADES 512/25)	VU
17	Fabaceae	*Myrocarpus frondosus* Allemao	yvyrá paje, ajuy ñandy	tree	bark	ST, SE-M, SR, SG-U	EN (MADES 512/25)	DD
18	Hypericaceae	*Hypericum connatum Lam.*	ka’avo tory	herb	flowering tops	SN, SD, SC, SE-M	EN (SEAM 1609/06). EN (MADES 512/25)	
19	Malvaceae	*Ceiba chodatii* (Hassl.) Ravenna	palo borracho, yuchan, samu’u, cucó	tree	prickles, thorns	SG-U, SM-E, SE-M, SD, ST	AE (MADES 512/25)	
20	Malvaceae	*Ceiba speciosa* (A. St.-Hil.) Ravenna	samu’u	tree	thorns and bark	ST, SE-M, SG-U	AE (MADES 512/25)	LC
21	Meliaceae	*Cedrela fissilis* Vell.	cedro	tree	bark, fruits	SE-M, ST	EN (MADES 512/25)	
22	Myrtaceae	*Eugenia uniflora* L.	ñangapiry	tree	fresh or dried leaves, bark	SC, SE-M, SD, SG-U, ST	VU (SEAM 1609/06)	LC
23	Myrtaceae	*Psidium grandifolium* DC.	katuáva guasu	shrub	leaves	SD, SG-U, SE-M, SN	EN (SEAM 1609/06), EN (MADES 512/25)	LC
24	Nymphaeaceae	*Victoria cruziana* Orb.	jakaré yrupé, maíz de agua	aquatic-marsh herb	flowers, seeds	SD, SN, SG-U	VU (SEAM 1609/06)	
25	Orchidaceae	*Cohniella jonesiana* (Rchb. f.) Christenson	suelda consuelda	epiphytic herb	whole plant	SG-U	EN (MADES 512/25)	
26	Orobanchaceae	*Escobedia grandiflora* (L. f.) Kuntze	ysypó ju, azafrán del pais, azafranillo	suffrutescent herb	root	ST, SE-M, SM-E	EN (MADES 512/25)	
27	Piperaceae	*Peperomia tetraphylla* (G. Forst.) Hook. & Arn.	ypeku ka’a	epiphytic herb	fresh plant	SD, SC	EN (MADES 512/25)	
28	Piperaceae	*Piper regnellii* (Miq.) C. DC.	jaguarundi	shrub	fresh leaves	SR, SD	EN (MADES 512/25)	
29	Pteridaceae	*Adiantopsis chlorophylla* (Sw.) Fée	doradilla crespa	herb	whole plant	SG-U, SD, SE-M	EN (MADES 512/25)	
30	Pteridaceae	*Hemionitis rufa* (L.) Sw.	doradilla	herb	whole plant	SG-U, SC, SR, SE-M	EN (MADES 512/25)	
31	Rutaceae	*Balfourodendron riedelianum* (Engl.) Engl.	guatambú	tree	bark	ST	EN (MADES 512/25)	EN
32	Simaroubaceae	*Picrasma crenata* (Vell.) Engl.	para’y, pyre’ro	tree	bark, wood (sawdust), root, leaves	SE-M, SG-U, ST	CR (SEAM 1609/06), EN (MADES 512/25)	
33	Zygophyllaceae	*Plectrocarpa sarmientoi* (Lorentz ex Griseb.) Christenh. & Byng	palo santo, guayacán, yvyrá okái	tree	bark, wood (sawdust)	ST, SD, SC, SR, SM-E, SG-U,	EN (SEAM 1609/06), EN (MADES 512/25)	EN

## Data Availability

The data presented in this manuscript are derived from the cited references. The results of the interviews are found in the tables available in the document.
